# Genome-wide characterization of C2H2 zinc-finger gene family provides insight into the mechanisms and evolution of the dehydration–rehydration responses in *Physcomitrium* and *Arabidopsis*

**DOI:** 10.3389/fpls.2022.953459

**Published:** 2022-10-03

**Authors:** Xuan Li, Xubing Cao, Jialing Li, Qinqin Niu, Yuanping Mo, Lihong Xiao

**Affiliations:** College of Forestry and Biotechnology, Zhejiang A&F University, Hangzhou, China

**Keywords:** dehydration-rehydration response, zinc-finger proteins, land plant evolution, desiccation tolerance, expression patterns

## Abstract

Dehydration tolerance is a vital factor for land plant evolution and world agricultural production. Numerous studies enlightened that the plant-specific C2H2-type zinc-finger proteins (C2H2-ZFPs) as master regulators played pivotal roles in the abiotic stress responses of plants. However, a comprehensive understanding of the evolution of C2H2-ZFPs in terrestrial plants and its regulatory mechanism in dehydration and rehydration response remains a mystery. In this study, the genome-wide identification of C2H2-ZFP genes revealed 549 homologs in the representatives of terrestrial plant lineages from liverwort to angiosperms. Based on the characteristics of the conserved C2H2-ZF domains, four major C2H2-ZF types (M-, Z-, Q-, and D-type) were identified in the C2H2-ZFPs, with the dominants of M-type in all selected species and followed by Z-type in non-seed plants and Q-type in seed plants, respectively. Phylogenetic analyses of the identified C2H2-ZFPs supported four major groups in the land plant representatives, among which the members from the desiccation-tolerant *Physcomitrium patens* and the dehydration-sensitive *Arabidopsis thaliana* displayed different topological relationships in the phylogenies reconstructed for a single species. C2H2-ZFPs clustered in the same subclades shared similar features in their conserved domains and gene structures. Approximately, 81% of the *C2H2-ZFP* promoters of all 549 identified C2H2-ZFPs harbored the conserved ABA-responsive elements (ABREs) and/or dehydration-responsive elements (DREs). Comparative transcriptomic analyses showed that 50 *PpZFPs* and 56 *AtZFPs* significantly changed their transcripts abundance. Interestingly, most of the dehydration- and rehydration-responsive *PpZPFs* and *AtZFPs* had been predicted to contain the ABRE and DRE elements in their promoter regions and with over half of which phylogenetically belonging to group III. The differences in the expression patterns of *C2H2-ZFPs* in responses to dehydration and rehydration between *P. patens* and *A. thaliana* reflected their different strategies to adapt to dehydration. The identified candidate *PpZFPs* were specifically induced by moderate dehydration and reached the peak transcript abundance in severe dehydration. Our study lays the foundations for further functional investigation of C2H2-ZFPs in dehydration responses from an evolutionary perspective in land plants. The findings will provide us with genetic resources and potential targets for drought tolerance breeding in crops and beyond.

## Introduction

The sessile lifestyle makes plants often suffer from various environmental stresses in nature. Among the abiotic stresses threatening global food security, drought is an important factor that hinders plant growth and crop yields worldwide by causing adverse cellular reactions, such as osmotic imbalance, endomembrane system damage, and declined respiratory and photosynthetic rates (Bartels and Sunkar, 2005; Liu et al., 2015; Zhu, 2016; Chen et al., 2018; Guo et al., 2018; Costa and Farrant, 2019; Han et al., 2020). In the past decades, the crop losses caused by drought stress have considerably increased because of global warming and changes in rainfall patterns (Riyazuddin et al., 2021). A recent survey indicated that the yields of major grain crops such as soybean, wheat, corn, and rice are all seriously affected by drought stress (Leng and Hall, 2019). A prediction shows that the level of food production must be at least 70% higher than the current level to meet the food demand of the world population by 2050 (Webber et al., 2018). Therefore, the identification and in-depth exploration of the functions of genes related to drought resistance or dehydration tolerance will hold the promise to improve crop resistance to drought and ultimately increase crop yields.

To counteract the effects caused by drought stress, plants have evolved cascade responses and protective mechanisms involving morphological, physiological, and molecular adaptations such as the expression of drought-related genes (Garg et al., 2002; Conditions et al., 2004; You et al., 2013; Lee et al., 2016; Han et al., 2020). Numerous previous studies suggested that transcription factors (TFs) as critical regulators involved in various biological and environmental stress processes through transcriptional regulation of downstream genes in plants (Wang et al., 2019; Han et al., 2020). The zinc-finger transcription factors (ZFPs), composed of at least one zinc-finger-binding domain and any of a variety of transcription factor effector domains, are known to be widely distributed in the genomes of all plant lineages (Yuan et al., 2018b). According to the number and position of cysteine (Cys) and histidine (His) residues, ZFPs were classified into C2H2, C4, C6, C4HC3, C3HC4, C2HC, C3H, and other types (Han et al., 2020). Among the ZFPs, the C2H2-type zinc-finger-containing proteins (C2H2-ZFPs), also known as the TFIIIA-type TFs, are currently reported as the most widely distributed and well-studied class of ZFPs in plants (Yuan et al., 2018b). The C2H2-ZFPs generally contain a specific conserved sequence composed of 25–30 amino acids bound to the Zn^2+^ atom and form a tetrahedral zinc-finger structure as C-X_2~4_-C-X_3_-P-X_5_-L-X_2_-H-X_3_-H, where X represents any amino acid (Han et al., 2020). It has been reported that most C2H2-ZFPs in plants contain a highly conserved QALGGH motif, which is also known as Q-type C2H2-ZFPs (Agarwal et al., 2007; Gourcilleau et al., 2011; Faraji et al., 2018).

Since the discovery of the first plant C2H2-ZFP EPF1 in *Petunia*, the C2H2-ZFPs have been systematically identified in many plants, such as the model plant *Arabidopsis thaliana*, some major and horticultural crops, and other economically important plants, such as rice, wheat, *Brassica rapa* L., soybean, cucumber, tomato, potato, cotton, and tea tree (Takatsuji et al., 1992; Englbrecht et al., 2004; Agarwal et al., 2007; Faraji et al., 2018; Yuan et al., 2018a; Alam et al., 2019; Liu Z. et al., 2020; Yin et al., 2020; Liao et al., 2021; Rehman et al., 2021; Zhang et al., 2021). Some identified C2H2-ZFPs have been reported, but only limited to several model plants, to participate in abiotic stress responses such as drought through transcriptional activation or inhibition (De Pater et al., 1996; Kiełbowicz-matuk, 2012; Han and Fu, 2019; Tang et al., 2019).

ZFP252 is the first C2H2-ZFP reported to be involved in drought stresses in the model monocot rice (Huang et al., 2005). The survival rate of ZFP252 overexpressing plants was significantly higher than that of wild-type and antisense-ZFP252 plants under drought stress (Huang et al., 2009). Overexpression of ZFP182 enhanced the tolerance to salt, drought, and cold stresses in rice plants (Huang et al., 2012), while ZFP36 plays a critical role in ABA-induced antioxidant defense and water stress tolerance (Zhang et al., 2014; Huang et al., 2018). Unlike the previously reported C2H2-ZFPs as transcriptional activators in rice, OsDRZ1 acting as a transcriptional repressor confers pleiotropic effects on rice growth and drought tolerance by downregulating stress-responsive genes (Yuan et al., 2018b). Interestingly, the enhanced stress tolerance was not accompanied by plant growth retardation in *OsDRZ1* transgenic plants, indicating the potential application of *OsDRZ1* in drought-tolerant rice engineering (Yuan et al., 2018b).

In *Arabidopsis*, overexpression of *ZAT10/STZ* and *ZAT12* enhanced tolerances to drought, high salinity, and cold but inhibited plant growth, evidenced by the induction of reactive oxygen species (ROS)-related genes (Rizhsky et al., 2004; Mittler et al., 2006). The knockdown mutants of ZAT18, a dehydration-induced C2H2-ZFP in *A. thaliana*, display reduced drought tolerance while the overexpression (OE) lines exhibit improved drought tolerance under drought treatment (Yin et al., 2017). Moreover, higher leaf water content, lower content of ROS, antioxidant enzyme activity, and overrepresentation of stress-responsive genes in *ZAT18* OE lines indicate that ZAT18 as a positive regulator plays a crucial role in the plant drought stress (Yin et al., 2017). Under dehydration stress, *AZF2* expression levels in *aba1* and *abi1* mutants were lower than those in the wild-type *Arabidopsis*, indicating that C2H2-ZPFs like *AZF2* enhance dehydration tolerance through an ABA-dependent pathway (Sakamoto et al., 2004).

In addition, studies on other plants have shown that some C2H2-ZFPs improve drought tolerance through multiple mechanisms other than the ABA-dependent or ABA-independent pathways. For example, transgenic *Arabidopsis* plants with *GmZFP3* showed enhanced expression of ABA-related marker genes, suggesting that GmZFP3 as a negative regulator participates in the tolerance of soybean to drought stress through the ABA-dependent signaling pathway (Zhang et al., 2016). Transgenic plants with *CgZFP1* (Gao et al., 2012) and *DgZFP3* (Liu et al., 2013) from chrysanthemum, *BcZAT12* from tomato (Rai et al., 2013), and *IbZFP1* from sweet potato (Wang et al., 2016) displayed improved drought resistance by increasing the levels of osmotic adjustment substances, improving ROS scavenging ability, and regulating downstream stress response genes. *Arabidopsis azf2* mutants overexpressing the *ThZF1* of *Thellungiella halophila* showed a flowering-time phenotype similar to that of the wild type under drought stress (Xu et al., 2007). *GsZFP1* overexpression in alfalfa enhanced drought tolerance by elevating the expression levels of stress-responsive marker genes, including *MtCOR47, MtRAB18, MtP5CS*, and *MtRD2* under drought stress (Luo et al., 2012).

Although some of the C2H2-ZFP members have been extensively studied in plants, there is still a lack of relevant knowledge about the evolution of C2H2-ZFPs in land plant lineages and their molecular functions in desiccation-tolerant plants, especially in non-seed plants. The model moss, *Physcomitrium patens*, as a member of the bryophytes of early differentiated land plant lineages, fills the gap between other green lineages such as aquatic algae and flowering plants (Gessel et al., 2017). The haploid gametophyte, a short growth cycle, high homologous recombination rate, simple development pattern, and haploid gametophyte life cycle make it a new model organism to gain insight into land plant evolution by comparative analyses with angiosperms (Komatsu et al., 2020). In particular, its ability to tolerate desiccation has attracted scientific attention across the global resource that could be applied in engineering drought-tolerant plants without yield penalty (Rensing et al., 2020; Upadhyaya et al., 2021). Our recent report suggests that gametophores, but not protonemata, can survive desiccation below −100 MPa after a gradual drying regime in an open system, without exogenous ABA (Xiao et al., 2018).

In this study, we identified not only the C2H2-ZFPs from the genomes of six representative land plant lineages, but also characterized their chromosomal location, gene structure, conserved motifs, promoters and reconstructed the phylogenetic relationships. To obtain the candidate key C2H2-ZFP genes from the desiccation-tolerant moss, we conducted comparative transcriptomic analyses during dehydration and rehydration of *P. patens* and *A. thaliana*. The *PpZFPs* that were induced by moderate dehydration and reached the highest transcript abundance in severe dehydration were identified as candidates for further crop breeding to increase crop yield under drought stress.

## Materials and methods

### Identification of C2H2-ZFP genes

The C2H2-ZFPs in *A. thaliana* and *O. sativa* had been previously identified in several studies but varied in the reported members (Englbrecht et al., 2004; Agarwal et al., 2007). To obtain the exact C2H2-ZFPs in *A. thaliana* and *O. sativa*, the amino acid sequences of all possible C2H2-ZFPs were retrieved from the TAIR (https://www.arabidopsis.org/) and Rice Genome Annotation Project (http://rice.uga.edu/index.shtml) websites, respectively. Then, the sequences were compared to the Swiss-Prot database (http://www.gpmaw.com/html/swiss-prot.html) with an expected value (*E*-value) of 1e-5. The obtained candidates were then submitted to the websites of InterProScan (http://www.ebi.ac.uk/interpro/) and NCBI Conserved Domain Data (CDD, https://www.ncbi. nlm.nih.gov/Structure/bwrpsb/bwrpsb.cgi) to verify the conserved C2H2 domains (IPR000690, IPR003604, IPR013085, IPR032553, IPR034736, IPR013087, IPR019406, IPR014898, IPR015880, and IPR007087), respectively. All of the candidates were manually inspected to confirm the presence of C2H2 zinc-finger domains using the Simple Molecular Architecture Research Tool (SMART: http://smart.embl.de/smart/batch.pl). Only candidates containing at least one of the conserved C2H2 domains were finally identified as C2H2-ZFPs in *A. thaliana* (158) and *O. sativa* (168) (Supplementary Table 1).

To identify the possible C2H2-ZFP genes in representatives of non-seed plants, BLASTP searches were performed against the genomes of *M. polymorpha, S. fallax, S. moellendorffii*, and *P. patens* with the *E*-value of 1e-5, respectively, using the full-length amino acid sequences of the newly identified C2H2-ZFPs in *A. thaliana* as queries (Supplementary Table 1). The obtained candidates were further verified using the same methods as the identification for *A. thaliana* and *O. sativa* above. All genome sequences and annotation files were downloaded from the Phytozome website (https://phytozome-next.jgi.doe.gov/) and the Ensemble database (http://plants.ensembl.org/info/data/ftp/index.html).

### Genomic distribution and gene duplication of C2H2-ZFPs

The genomic locations of all identified C2H2-ZFPs were retrieved from the gff files of the selected species and visualized by the MG2C (http://mg2c.iask.in/mg2c_v2.1/). The graphs were manually adjusted to reflect the descending order of chromosome or scaffold lengths. Meanwhile, the orthologous and homoeologous gene pairs of C2H2-ZFP genes were determined within species according to the physical position on chromosomes and sequence similarity. The syntenic relationships of C2H2-ZFP gene pairs within and between species were displayed as circular diagrams using TBtools software. The ratio of non-synonymous to synonymous nucleotide substitutions (*Ka*/*Ks*) was evaluated among segmentally duplicated gene pairs to detect the selection mode by TBtools software.

### Characteristics, gene structure, and conserved motif

The molecular weight (MW), isoelectric point (pI), and grand average of hydropathicity (GRAVY) of the identified C2H2-ZFPs were investigated by the online program ExPASy (http://web.expasy.org/protparam/). Subcellular localization for each C2H2-ZFP was predicted by CELLO v2.5 online (http://cello.life.nctu.edu.tw/).

The gene structures of C2H2-ZFPs were analyzed and visualized by GSDS (http://gsds.gao-lab.org/) based on the genomic features defined in the gff files.

### Classification of C2H2-ZFPs

The C2H2-ZF types of the identified C2H2-ZFPs were determined according to the plant-specific amino acid residues and distances between two to more C2H2 zinc-finger domains, as previously reported in soybean (Yuan et al., 2018b). All C2H2-ZFPs were analyzed using the SMART database combined with a manual inspection to confirm the numbers of C2H2-ZF domain, amino acid sequences of C2H2-ZF domain, and the space length between two adjacent C2H2-ZFs. Based on the description of Yuan et al. (2018b), the identified C2H2-ZFPs were divided into four types, including Q-type, M-type, Z-type, and D-type. In more detail, C2H2-ZFPs containing a plant-specific conserved amino acid motif “QALGGH” and a conserved spacing “X_2_-C–X_2_-C–X_7_-QALGGH–X_3_-H” in the C2H2-ZF domains were classified as Q-type; those containing one to five degraded amino acids in the “QALGGH” motif and certain modifications in the spacing between two cysteines (C2) and two histidines (H2) were designated as M-type (M1~M5). The Z-type was characterized by the C2H2-ZF domains with more than 12 (Z1) and < 12 (Z2) in their spacing between the second cysteine and the first histidine. The D-type did not include the second histidine in the C2H2-ZF domain compared with the other three types.

### Promoter *cis*-acting elements in promoters of *C2H2-ZFPs*

The 2,000 base pair (bp) DNA sequences upstream of ATG of the *C2H2-ZFPs* were retrieved from the reference genomes and submitted to the PlantCare database (http://bioinformatice.psb.ugent.be/webtools/plantcare/) to identify the putative cis-regulatory elements.

### Sequence alignment and phylogenetic analysis of C2H2-ZFPs

To comprehensively understand the evolutionary relationship of the C2H2-ZFPs among land plant lineages, multiple sequence alignments were performed using all conserved domains of the 549 identified C2H2-ZFPs and a C2H2-ZFP Cre13.g567250 of *Chlamydomonas reinhardtii* (as outgroup) by MAFFT (https://mafft.cbrc.jp/alignment/server/). The aligned sequences were used to construct the phylogenetic relationship with the maximum likelihood (ML) method (bootstrap = 1,000 and default parameters) by iqtree 1.6.12 software (Nguyen et al., 2015).

The unrooted phylogenetic trees of PpZFPs of the desiccation-tolerant moss *P. patens* and AtZFPs of the dehydration-sensitive angiosperm *A. thaliana* were reconstructed, respectively, using the procedures above. All the phylogenetic trees were further visualized by iTOL (https://itol.embl.de/).

### Expression analysis of *PpZFPs* and *AtZFPs* during dehydration and rehydration

To explore the roles of C2H2-ZFPs in response to the dehydration and rehydration between vegetative tissues of the basal and dicot land plants, comprehensive expression profiles of C2H2-ZFPs of *P. patens* (6-week-old gametophores) and *A. thaliana* (2-week-old seedlings) were performed using our previous RNA-seq data, representing five stages of dehydration and rehydration with three biological bio-replicates, including hydrated control (HD), moderate dehydration (MDH), severe/deep dehydration (SDH/DDH), partial rehydration (PRE), and full rehydration (FRW) during the dehydration and rehydration (Supplementary Table 2). For both species, HD represented the hydrated control, and MDH, PRE, and FRE indicated the relative water content (RWC) of 70, 50, and 100%. For SDH/DDH treatment, it represented 10% RWC for the desiccation-tolerant *P. patens* and 30% RWC for the desiccation-sensitive *A. thaliana*. The RNA-seq data were generated on Illumina HiSeq 2,000 platform using 6-week-old gametophores of *P. patens* and 2-week-old seedlings of *A. thaliana*. Samples with three biological replicates were collected for each of the five sampling stages. The dehydration and subsequent rehydration treatment and the measurement of RWC of *P. patens* were performed as described by Xiao et al. (2018). The dehydration and rehydration treatment and determination of RWC of *A. thaliana* followed the description of Xiao et al. (2015).

The transcriptomic datasets were deposited in the GEO database of NCBI (accession no. PRJNA800025). Clean data were obtained by removing the low-quality reads (with adaptors only and unknown bases more than 10%, and with more than 50% of the bases' quality value lower than in a read) using PRINSEQ v0.20.374 (http://prinseq.sourceforge.net/). High-quality clean reads were then mapped to the reference genomes (https://phytozome.jgi.doe.gov/pz/portal.html) using HISAT2 v2.0.975 (http://daehwankimlab.github.io/hisat2/).

To obtain high confident results, three packages, Cuffdiff v2.1.1 (Trapnell et al., 2013), DESeq 1.18.0 (Anders and Huber, 2010), and edgeR 3.36.0 (Robinson et al., 2009) were employed to determine the gene expression levels and the differentially expressed genes (DEGs). Only those genes with at least twofold change between the hydrated control and the de- or rehydration treatment with the threshold of *q*-value (Cuffdiff2), adjusted *p*-value (DESeq), and FDR (edgeR) < 0.05 at the same time were assigned as DEGs. All the differentially expressed C2H2-ZFP genes in *P. patens* and *A. thaliana* were filtered out from the DEGs, and the expression patterns and heatmaps of the differentially expressed *C2H2-ZFPs* were generated and visualized through the software Multiple Experiment Viewer v.4.9 (MEV, Williams et al., 2019).

### Preparation of plant material and transient subcellular localization of two selected PpZFPs

To verify the property as transcription factors of the key dehydration-related PpZFPs, the wild-type *P. patens* strain “Gransden 2004” preserved in the laboratory was used to obtain the coding sequences of two PpZEP genes, *Pp3c7_7980* and *Pp3c20_14750*. Subcultures of *P. patens* protonema were grown on solid mediums of BCDATG overlaid with cellophane disks (protonemata) or BCD (gametophores) in plastic Petri dishes (9 cm diameter), respectively. The plates were placed in an incubator (Percival CU36L6, Perry, IA, USA), with a light intensity of 50 μmol m^2^ s^−1^ delivered in a 16/8-h light/dark cycle at 23 ± 1°C.

Total RNA was isolated from 30 days old gametophores of *P. patens* using TRIzol reagent (Invitrogen, China), and the cDNA was generated using HiScriptII 1st Strand cDNA Synthesis Kit (Vazyme, China). The gene-specific primers of the two selected PpZFPs that were specifically induced or repressed by dehydration and/or rehydration were designed based on the CDS sequences of the genes by multiple primers analyzer from the website of ThermoFisher (https://www.thermofisher.cn/cn/zh/home/brands/thermo-scientific/molecular-biology/molecular-biology-learning-center/molecular-biology-resource-library/thermo-scientific-web-tools/multiple-primer-analyzer.html). The full-length coding sequences without stop codon from two representative dehydration-induced PpZFP genes were cloned into the modified pCAMBIA1300 vector by restriction enzyme digestion-DNase ligation cloning, respectively. Each CDS was fused into in-frame to the N-terminal of the green fluorescent protein coding sequence without the start codon at *SacI*/*XbaI* (*Pp3c7_7980*) and *KpnI*/*XbaI* (*Pp3c20_14750*) restriction sites under the control of the CaMV35S promoter, respectively (Supplementary Figure 1). The primers for each of the genes were listed in Supplementary Table 3. Both the fusion vectors (containing 35S::PpZFPs-GFP) and the control vector (containing 35S::GFP only) were transformed into protoplasts of *P. patens* by PEG-mediated transformation (Pu et al., 2020). The transformed protoplasts were incubated on ABCG liquid medium at 25°C for 20 h, and the GFP fluorescence was examined at 488 nm by a laser scanning confocal microscope (TCS SP8, Leica, Germany). All of the vectors and constructs used in this study were extracted and purified using the Simgen Plasmid Extract Kit (Simgen, China).

## Results

### Identification of C2H2-ZFPs in the representatives of land plant lineages

Integrating the validations of online programs of Swiss-Prot, NCBI CDD and InterProScan and SMART and manually inspection in this study with the previous reports (Englbrecht et al., 2004; Agarwal et al., 2007; Chen et al., 2020a), we confirmed 168 and 158 C2H2-ZFPs in the genomes of *O. sativa* and *A. thaliana*, respectively. The possible C2H2-ZFPs in *M*. *polymorpha, S. moellendorffii, S*. *fallax*, and *P. patens* were obtained by a two BLAST method using the protein sequences of the identified C2H2-ZFPs in *A. thaliana* as queries. With the same methods for identification of *O. sativa* and *A. thaliana*, 17, 53, 41, and 112 C2H2-ZFPs were confirmed in *M*. *polymorpha, S. moellendorffii, S*. *fallax*, and *P. patens*, respectively. In total, 549 C2H2-ZFPs were detected and verified in the six representative land plant lineages (Table 1; Supplementary Table 1). For the genes with several isoforms, we used the amino acid sequences that were annotated as primary isoforms in their annotation files for further analyses.

**Table 1:** Overview and classification of C2H2-ZFPs in the representatives of land plant lineages (including statistics of C2H2-ZFPs with only C2H2-ZF domain(s) in each species).

**Species**	***A. thaliana***	***O. sativa***	***P. patens***	***S. moellendorffii***	***S. fallax***	***M. polymorpha***	**Total**
No. of C2H2-ZPFs	158	168	112	53	41	17	549
No. of C2H2-ZFPs with only C2H2-ZF domain(s)	108	136	63	38	19	14	378

### Global view of C2H2-ZFP genes in the representatives of land plant lineages

The physical maps of C2H2-ZFP genes displayed the scattered distribution patterns in the genomes of six representative species (Supplementary Figures 2–7). In detail, all five chromosomes of *A. thaliana* harbored the *C2H2-ZFPs* (*AtZFPs*), over half of which were distributed on the chromosomes of 5 (46 *AtZFPs*) and 1 (37 *AtZFPs*) (Supplementary Figure 2). The *C2H2-ZFPs* of *O. sativa* (*OsZPFs*) were mapped on 12 chromosomes, of which the chromosome 3 contained the most *OsZPFs* (24), followed by chromosome 1 (21) (Supplementary Figure 3). The 112 *C2H2-ZFPs* in *P. patens* (*PpZFPs*) were scattered on 27 chromosomes, with one to eight on each chromosome (Supplementary Figure 4). Of the 41 *C2H2-ZFPs* in *S. fallax* (*SfZFPs*), 40 were distributed on 17 of 19 chromosomes and only one was located on scaffold 50 (Supplementary Figure 5), while all 17 and 53 *C2H2-ZFPs* in *M. polymorpha* (*MpZFPs*) and *S. moellendorffii* (*SmZFPs*) were evenly distributed on 16 and 37 scaffolds with one or two genes for each scaffold (Supplementary Figures 6, 7). Moreover, there were 11, 15, and 5 tandem repeat events referring to 26, 34, and 12 *C2H2-ZPFs* in *A. thaliana, O. sativa* and *P. patens*, but only one event involved in one pair of *C2H2-ZFPs* in *S. moellendorffii* and *S*. *fallax*, respectively (Supplementary Figures 6, 7).

**Figure 1:**
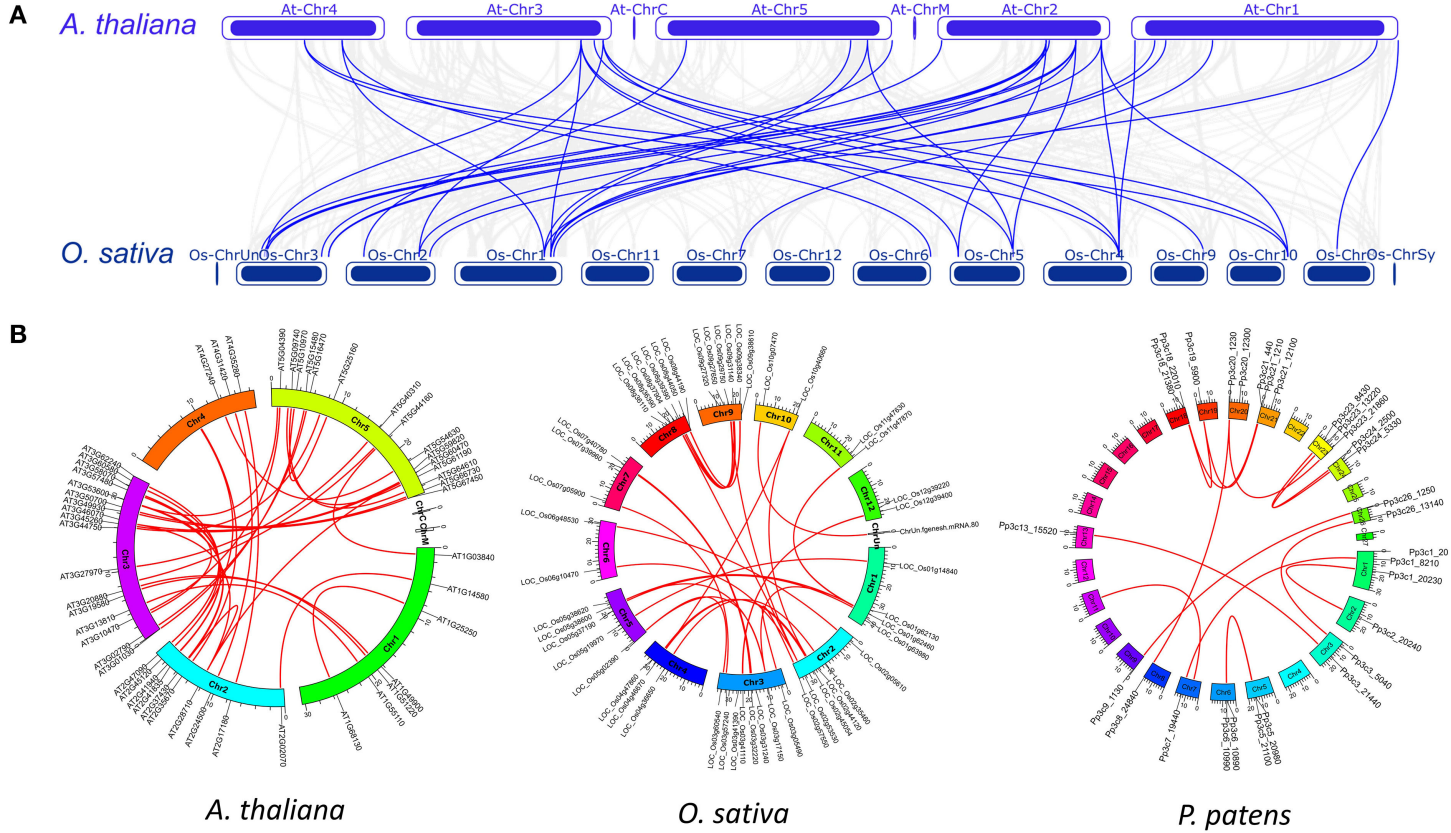
Interspecific and intraspecific syntenic relationships of *C2H2-ZFPs* in the representatives. **(A)** Syntenic relationships of *C2H2-ZFPs* between *A. thaliana* and *O. sativa*. Gray lines in the background indicate the genome-wide syntenic blocks while red lines highlight the syntenic gene pairs of C2H2-ZFPs. **(B)** Syntenic relationships of *C2H2-ZFPs* in *A. thaliana, O. sativa* and *P. patens*. Gray lines in the background indicate the genome-wide syntenic blocks while red lines highlight the syntenic gene pairs of *C2H2-ZFPs*.

The syntenic analysis showed that the interspecies synteny of *C2H2-ZFPs* was only identified between *O. sativa* and *A. thaliana* (36) among the six species (Figure 1A; Supplementary Table 4). Intraspecies syntenic analysis revealed 27 (30.2%), 29 (34.5%), and 11 (19.6%) gene pairs of *C2H2-ZFPs* in *A. thaliana, O. sativa*, and *P. patens*, respectively (Figures 1B–D; Supplementary Table 4). No intraspecies synteny was detected for *C2H2-ZFPs* in *M*. *polymorpha, S. moellendorffii*, and *S*. *fallax*. The results from genomic localization and synteny of *C2H2*-ZFPs implied that whole-genome duplication events were the main mechanism of the expansion of C2H2-ZFP genes in the representative species.

To have an idea about the selective pressure of the paired *C2H2-ZFPs* with intraspecific syntenic relationships, the *Ka/Ks* ratio was calculated by TBtools suite package. The *Ka/Ks* ratios of *PpZFPs* and *AtZEPs* ranged less than 0.33 (Supplementary Table 4), indicating that purification selection (negative selection) occurred during their evolution. However, the *Ka/Ks* ratios of the *OsZFPs* in colinear gene pairs range from 0.13 to 0.94 (Supplementary Table 4), reflecting the relatively rapid evolution of *OsZFPs*. Three pairs of *OsZFPs* showed over 0.92 *Ka/Ks* ratios implying that frequent synonymous substitutions occurred in these genes. These results signified the different evolutionary rates of *C2H2-ZFPs* during species evolution.

### General characteristics of C2H2-ZFPs of the representative species

To comprehensively characterize the identified C2H2-ZFPs of the land plant representatives, the basic information of the genes including the lengths of amino acid sequence (aa), molecular weight (MW), isoelectric point (pI), grand average of hydropathicity (GRAVY), subcellular localization prediction, and classification were analyzed (Supplementary Table 5).

The lengths of C2H2-ZFPs amino acid sequences varied within and among the analyzed species, with an average length ranging from 353 aa (SmZFPs) to 793.83 aa (SfZFPs), of which 110103 of *S. moellendorffii* (56 aa) was the shortest and Mapoly0003s0206 of *M*. *polymorpha* (2,385 aa) was the longest (Supplementary Table 5). Accordingly, the analyses of physicochemical properties of the identified C2H2-ZFPs showed a range of variation in MWs, ranging from 6.06 kDa for 110103 of *S. moellendorffii* to 261.16 kDa for Mapoly0003s0206 of *M*. *polymorpha* and pI values (4.27 for Pp3c2_6080 of *P. patens* to 10.14 for 56696 of *S. moellendorffii*), respectively (Supplementary Table 5). Except for the LOC_Os01G32920 (0.029) of *O. sativa*, the predicted GRAVY values of all C2H2-ZFPs were negative, suggesting the hydrophilic nature of the proteins (Supplementary Table 5).

Subcellular localization prediction showed that the 534 C2H2-ZFPs could be located in the nucleus, and 15 exceptions could be located in the chloroplast (2), mitochondrion (1), extracellular (4), cytoplasmic (7), or plasma membrane (1) (Supplementary Table 5).

### Gene structure, conserved motifs, and classification of C2H2-ZFPs of the representative species

Analyses of exon–intron organization of the C2H2-ZFP genes exhibited high heterogeneity among and within species (Table 1; Supplementary Figures 8–13). Of the identified *C2H2-ZFPs*, the number of genes without intron and the maximum number of introns in a single gene increased with the complexity of the organisms with 1 (*MpZFPs* and *SfZFPs*), 10 (*PpZFPs*), 18 (*SmZFPs*), 68 (*AtZFPs*), and 74 (*OsZFPs*) intron-less *C2H2-ZFPs* and 10–20 maximum introns in each species (Table 1; Supplementary Figures 8–13).

To reveal the diversification of the identified C2H2-ZFPs, the full-length protein sequences of each C2H2-ZFP were inspected for all known conserved domains. A total of 60 other known domains besides the C2H2-ZF domains were predicted (Supplementary Table 6). Based on the presence or not of these additional domains and their organization, a total of 378 C2H2-ZFPs (378/549 = 68.9%) in the representative species were identified as the first major group containing only C2H2-ZF domain(s), with varied members among species (Table 1; Supplementary Table 6). Fifty members (50/549 = 9.1%) containing a coiled-coil domain besides the C2H2-ZF domain(s) were ranked the second major group (Supplementary Table 6). The third major group included 29 C2H2-ZFPs that harbored one or more additional zinc ion-binding domain(s), such as RING, AN1, Znf UBA, Znf C4HC3, besides the C2H2-ZFs (Supplementary Table 6), while the rest groups included at least one additional known domain that was predicted to be involved in various molecular functions (Supplementary Table 6).

**Figure 2:**
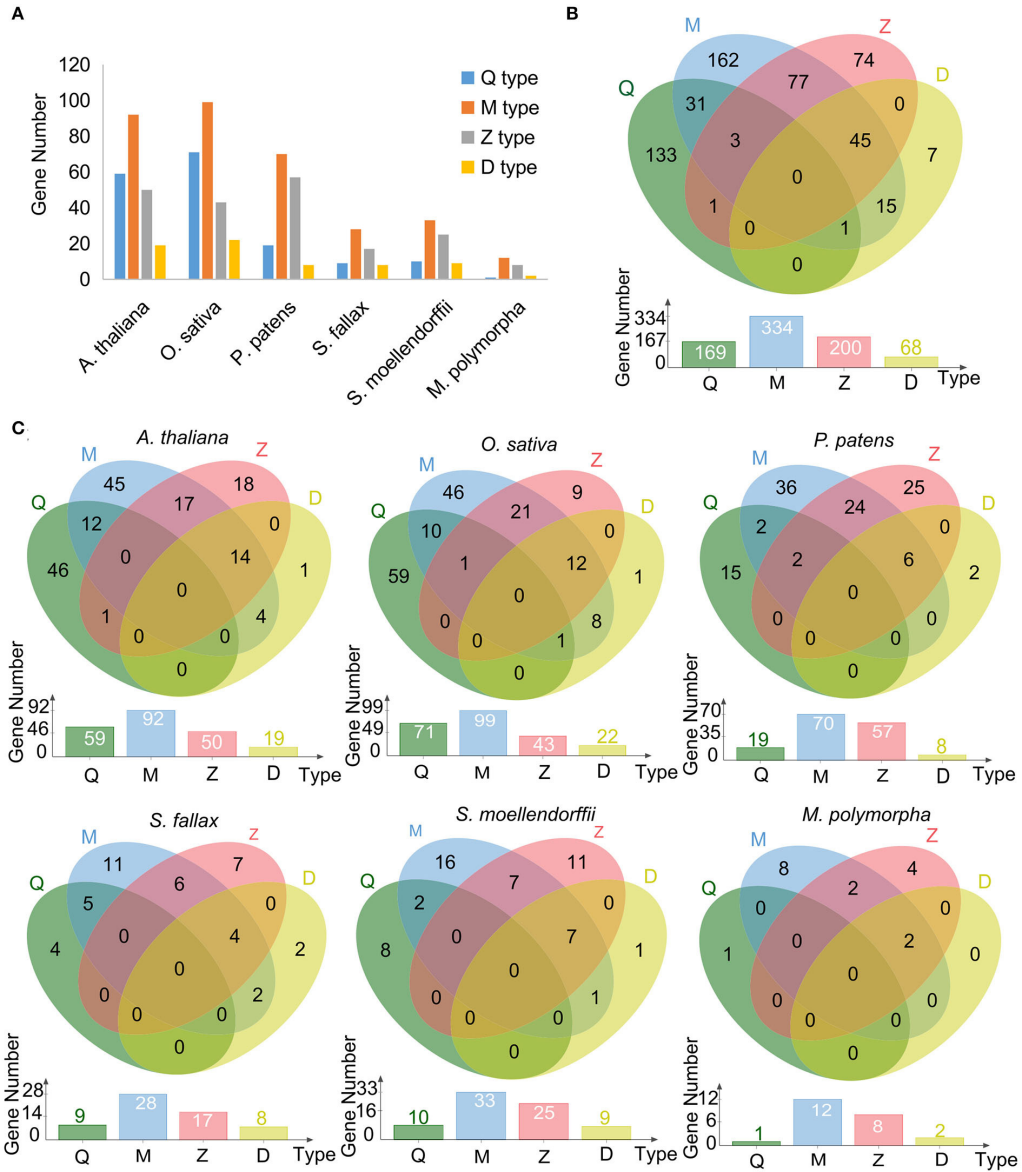
Subset classification of C2H2-ZFPs in the representatives of land plant lineages. **(A)** Bar diagram summarizes the distribution of each type of C2H2-ZF domains in six representative land plant species. **(B)** Venn diagram summarizes the distribution of each type of C2H2 domains in six representative land plant species. **(C)** The detailed distribution of each C2H2-ZF domain in each representative.

A total of 1,087 C2H2-ZF domains for the 549 C2H2-ZFPs were identified with 1–9 C2H2-ZF domain(s) for each of the proteins (Supplementary Table 6). The C2H2-ZFPs were further classified into four types, named Q-, M-, Z-, and D-type, respectively, based on the variation of the plant-specific conserved amino acid sequence “QALGGH” and distances between metal ligands in the C2H2-ZFs (Figures 2A–C; Table 1; Supplementary Table 6). Among the types, M-type C2H2-ZFs (including M1–M5) represented the largest member of C2H2-ZFPs in all representative species, followed by Q-type in two angiosperm species, or Z-type in three non-seed plant species (Figures 2A–C; Table 1). The D-type represented the least number of categories among species besides *M. polymorpha*. Moreover, 173 of the 549 C2H2-ZPFs had two or three of the types at the same time (assigned as mixed types here), and the remaining 376 C2H2-ZPFs belonged to only one of the types with 283 of which containing a single C2H2-ZF domain (Figure 2B; Supplementary Table 6).

### Promoter analyses of *C2H2-ZFPs* among the representatives of land plant lineages

**Figure 3:**
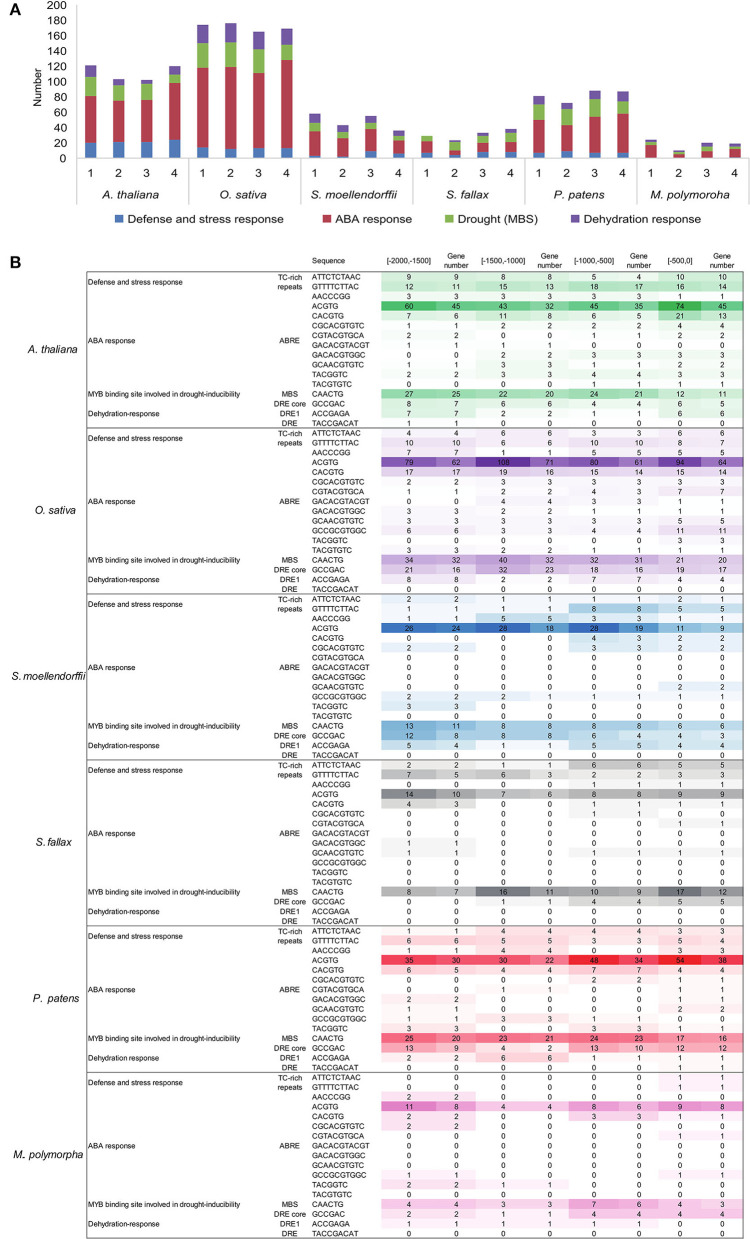
Promoter analyses of *C2H2-ZFPs* in the representatives of land plant lineages. Drought, dehydration, defense, and ABA-related cis-acting elements were counted for 2000 bp upstream from ATG. **(A)** The number of the drought, dehydration, defense, and ABA-related cis-acting elements in the *C2H2-ZFP* promoter region of each representative. **(B)** The details of the of drought, dehydration, defense, and ABA-related cis-acting elements in the *C2H2-ZFP* promoter region of each representative.

To better understand the transcriptional regulation of the *C2H2-ZFPs*, 2,000-bp promoter sequences upstream from ATG in the 549 *C2H2-ZFPs* were examined for the presence of *cis*-acting elements. Results revealed that the promoter regions of all *C2H2-ZFPs* had various *cis*-regulatory elements, except for the core elements such as CAAT-box, CCAAT-box, and TATA-box (Supplementary Table 7). For example, multiple *cis*-regulatory elements responsive to phytohormones, such as the abscisic acid-responsive elements (ABREs), auxin-responsive element (TGA-box), and MeJA-responsive elements (CGTCA), were identified in the *C2H2-ZFP* promoters of all the representative species (Supplementary Table 7). The detailed analysis revealed that the stress-associated *cis*-acting elements, such as the low-temperature-responsive (LTR) elements, MYB binding site involved in drought inducibility (MBS), dehydration-responsive elements (DREs), defense and stress responsiveness (TC-rich repeats), were also identified in the promoters of *C2H2-ZFPs* of the representatives of land plant lineages (Figure 3; Supplementary Table 7). The predicted elements were unevenly distributed in the promoter regions of the *C2H2-ZFPs*, but the distribution patterns showed similar trends among species (Figures 3A,B; Supplementary Table 7). Among them, the ABRE- and/or DRE-related elements, functioning in ABA and dehydration responses, represented the dominant *cis*-regulators of *C2H2-ZFP* promoters in the representative species with ranges from 69.6 to 94.1% (Figure 3; Supplementary Table 7). The ABRE elements especially the ACGTG motif sequence represented the most abundant *cis*-regulatory elements in the majority of the *C2H2-ZFP* promoters from all six species (Figures 3A,B; Supplementary Table 7). An MBS element with the motif sequence of CAACTG was listed as the second one, followed by elements function in defense and stress response, and dehydration response (Figures 3A,B; Supplementary Table 7).

### Phylogenetic relationship among C2H2-ZFPs

**Figure 4:**
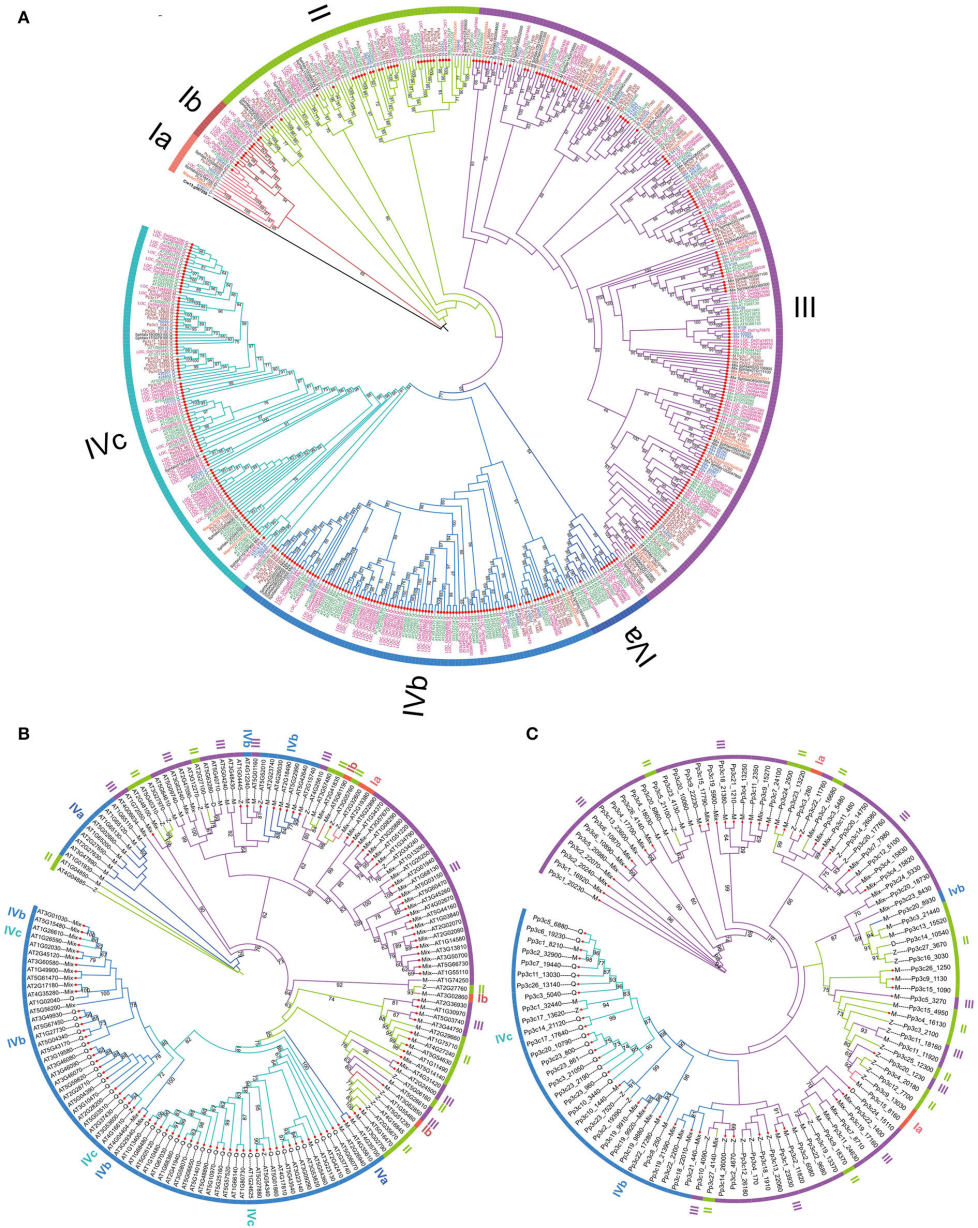
Phylogeny of C2H2-ZFPs. **(A)** Phylogeny of 549 identified C2H2-ZFPs. **(B,C)** Phylogeny of PpZFPs and AtZFPs. The trees were constructed by iqtree 1.0 software using maximum likelihood (ML) method with 1,000 bootstraps. The C2H2-ZFPs containing only C2H2 domain are marked with small red circles.

To evaluate the phylogenetic relationships among the C2H2-ZFPs in the representatives of land plants, a maximum likelihood (ML) tree was constructed using the concatenated amino acid sequences of all predicted C2H2-ZFs from each of the identified C2H2-ZFPs with a *C. reinhardtii* C2H2-ZFP (Cre13.g567250) as root (Supplementary Table 8). The phylogenic tree classified the C2H2-ZPFs into four major groups with 25, 76, 219, and 230 members, respectively (Figure 4A; Table 2). Group I represented the ancestral homologs because it contained a C2H2-ZFP from the aquatic green algae *C. reinhardtii*. The phylogenetic topology of group I can be further divided into two subgroups: Ia (13 members) and Ib (12 members) (Figure 4A; Supplementary Figure 14). Subgroup Ia included one to three homologs from the six representative species and the root homolog of *C. reinhardtii*, while subgroup Ib represented the angiosperm-specific expansion with 11 of 12 members containing no additional conserved motif besides C2H2-ZFs for two representative seed plants—three from *A. thaliana* and nine from *O. sativa* (Table 2). The C2H2-ZFPs in group II included members from all six species, and the majority of the members (except for seven members) harbored only one type of the conserved C2H2-ZF domain(s) (Figure 4A). Approximately, 65% (142) of the 219 C2H2-ZFPs in group III contained no additional conserved motif besides, and nearly two-third of the members had mixed C2H2-ZF types. Group IV included the most C2H2-ZFPs (41.9%) with 198 of which contained no additional conserved motifs besides C2H2-ZF domain(s) (86.1%) (Figure 4A; Table 2). Interestingly, over 57.8% C2H2-ZFPs in group IV contained Q-type C2H2-ZF domain(s) only, which did not exist in the members of the other three groups (Figure 4A). Among the four groups, members of groups III and IV displayed extreme expansion with 74–85% of the members in each species, and group IV included over 50% C2H2-ZFPs in the two seed plant representatives followed by group III, while more members of the group III in non-seed plant representatives (Table 2). According to the phylogenetic relationships, we further divided the group into three subgroups – IVa (18), IVb (102), and IVc (110) (Figure 4A; Table 2). The subgroup IVa represented an angiosperm-specific expansion of C2H2-ZFPs with 11 from *A. thaliana* and seven from *O. sativa*, while 78 (74.5%) of the members in IVb and 73 (66.4%) in IVc belonged to angiosperm-specific expansion of C2H2-ZFPs, respectively (Figure 4A; Table 2). In addition, all the Q-type C2H2-ZF domain-containing C2H2-ZFPs were clustered into the subgroups IVb and IVc (Figures 4A–C; Table 2).

**Table 2:** Segmental duplication gene pairs in *P. patens* and *A. thaliana*.

**Group**	**Subgroup**	**Gene number**	***A. thaliana***	***O. sativa***	***P. patens***	***S. fallax***	***S. moellendorffii***	***M. polymorpha***
I		24	5	10	3	2	3	1
	Ia	12	2	1	3	2	3	1
	Ib	12	3	9	0	0	0	0
II		77	22	23	20	6	5	1
III		218	48	51	57	22	28	12
IV		230	83	84	32	11	17	3
	IVa	18	11	7	0	0	0	0
	IVb	102	38	38	13	5	7	1
	IVc	110	34	39	19	6	10	2
Total		549	158	168	112	41	53	17

The *Ka/Ks* (the ratio of non-synonymous to synonymous nucleotide substitutions) were evaluated among segmentally duplicated gene pairs to detect the selection mode.

The phylogenetic topologies and relationships of AtZFPs and/or PpZPFs displayed significant differences from those constructed using 549 identified C2H2-ZFPs of the land plant representatives (Figures 4B,C; Supplementary Figures 15, 16; Supplementary Table 9). AtZFPs or PpZFPs that clustered into the same group in the phylogenetic tree of all 549 identified C2H2-ZFPs demonstrated chimeric distribution in the trees containing (Figures 4B,C; Supplementary Figures 15, 16; Table 2; Supplementary Table 9). In comparison, the *A. thaliana* genome was more abundant in the group IV AtZFPs than the *P. patens* genome, which encoded more members of the group III PpZFPs (Table 2). Besides, the Q-type C2H2-ZF domain(s)-containing AtZPFs belonged to the subgroups IVb and IVc, and they were clustered into a single subclade (Figure 4B; Supplementary Figure 15), but all Q-type C2H2-ZF domain(s)-containing PpZPFs were included in the subgroup IVc and clustered together (Figure 4C, Supplementary Figure 16).

### Expression profiles of the *PpZFPs* and *AtZFPs* in responses to dehydration and rehydration

**Figure 5:**
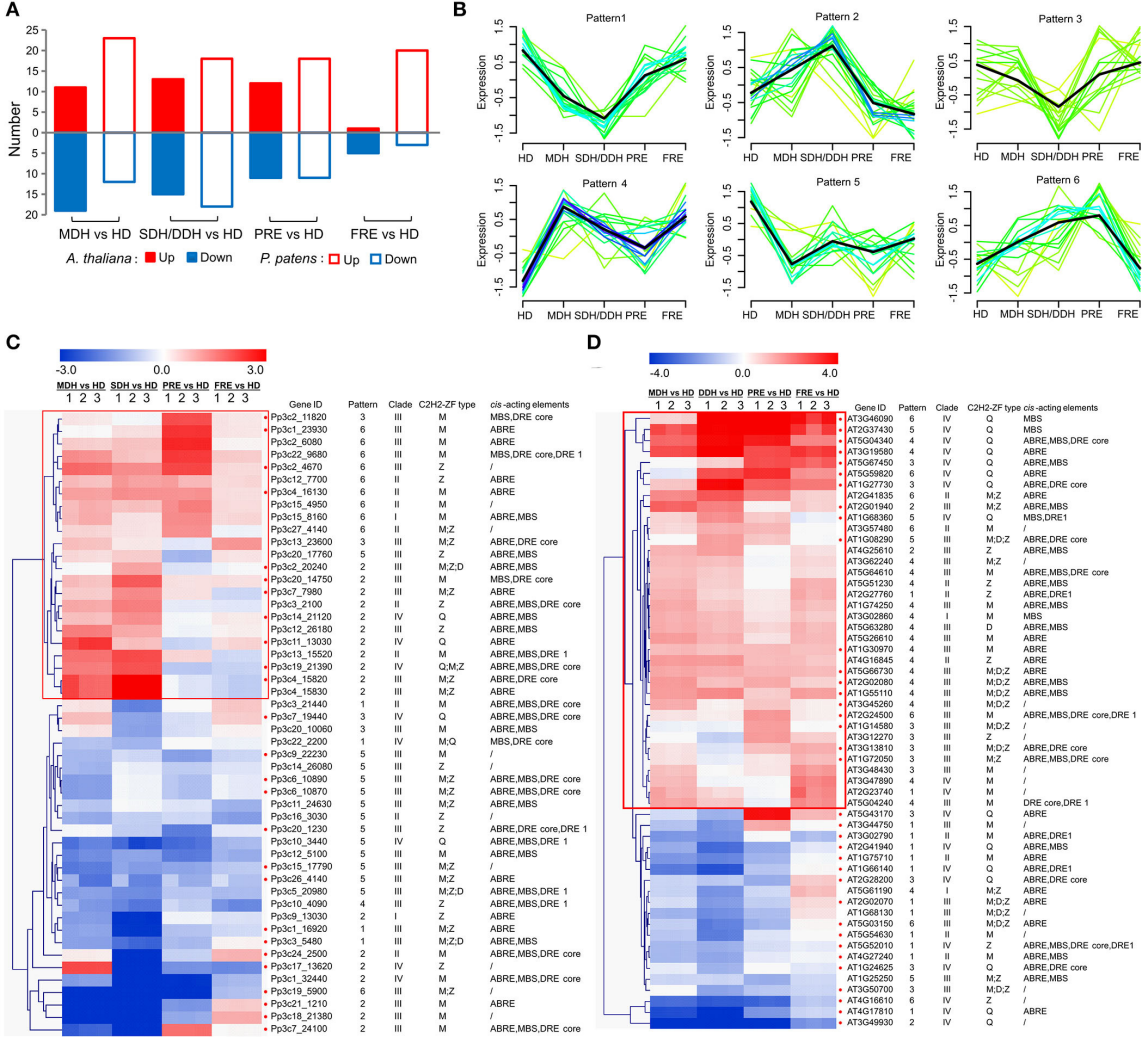
Profiles of the differentially expressed *PpZFPs* and *AtZFPs* in the responses of dehydration and rehydration. **(A)** All differentially expressed genes and differentially expressed *PpZEPs* and *AtZFPs*. **(B)** Profiles of the differentially expressed *AtZEPs*. **(B)** Mfuzz clustering of the differentially expressed *PpZEPs* and *AtZEPs*. **(C)** Integrated profile of the differentially expressed *PpZEPs*. **(D)** Integrated profile of the differentially expressed *AtZEPs*. In the color panels, each horizontal line represents a single gene, and the color of the line indicates the expression level (in a log scale) of the gene.

Based on our RNA-sequencing data generated previously from the samples of *P. patens* and *A. thaliana*, representing hydrated control (HD), moderate (MDH), and severe or deep dehydration (SDH or DDH), partial and full rehydration (PRE and FRE), we investigated the expression profiles of the *PpZFPs* and *AtZFPs*. A total of 217 *C2H2-ZFPs* (96 *PpZFPs* and 121 *AtZFPs*) were detected transcripts in at least one sampling point during the dehydration and rehydration in the two species, respectively, of which 50 *PpZFPs* and 56 *AtZEPs* were identified as the differentially expressed genes (DEGs) (Figure 5A; Supplementary Tables 1–10). Over two-third of the DEGs contained M-type C2H2-ZF motif(s) in their encoded proteins, while the proteins encoded by the differentially expressed PpZFPs containing Z-type C2H2-ZF domain(s) accounted for half of the total in *P. patens* (Figure 5C; Supplementary Table 10). Similar trends were found in *A. thaliana* with a relatively small proportion of AtZFPs containing M-type (~60%) and Z-type (~40%) C2H2-ZF domains (Figure 5D; Supplementary Table 10). Moreover, almost all the M-type and Z-type C2H2-ZF domain-containing DEGs were phylogenetically in clade III (Figures 5B,C). A total of 7 Q-type PpZFP genes and 15 Q-type AtZFP genes significantly changed their transcript abundance during dehydration and rehydration (Figures 5B,C). All Q-type C2H2-ZF domain-containing DEGs were ranked as the third and phylogenetically belonging to clade IV in both species (Figures 5B,C).

The Mfuzz clustering of the DEGs revealed six expression patterns with the DEG number ranging from 17 to 21 (Figure 5B; Supplementary Table 10. In detail, DEGs in patterns 1 and 3 exhibited similar expression trends with the peak expression levels in the samples of hydrated control and full dehydration, and meanwhile, the number of *AtZFPs* far exceeds the number of *PpZFPs* (over six times). DEGs in pattern 2 had the peak transcripts abundance in the samples of SDH (*P. patens*) or DDH (*A. thaliana*) with the ratio of *PpZFPs*: *AtZFPs* equal to 6:1. Pattern 4 included only one DEG of *PpZFPs* and 18 DEGs of *AtZFPs*, which had the highest transcripts abundance in the MDH samples, while the DEGs in pattern 5 contained four *AtZFPs* and 13 *PpZFPs* with the highest transcription abundances in the hydrated controls. Pattern 6 represented those *C2H2-ZFPs* (7 *AtZPFs* and 10 *PpZFPs*) that highly accumulated their abundances during the early stage of rehydration (PRE) in both species.

To comprehensively elucidate the functions of *C2H2-ZFPs* in responses to dehydration and rehydration, the differentially expressed *AtZPFs* and *PpZFPs* were further displayed by integrating information of transcripts abundance, evolutionary relationship, C2H2-ZF types, and cis-elements in the promoters of the differentially expressed *PpZFPs* and *AtZFPs* (Figure 5C; Supplementary Table 10). Eleven of the induced *PpZFPs* specifically accumulated transcripts during dehydration and reached peak abundance at the SDH stage and shared the same expression pattern (Pattern 2) and *cis*-acting elements, ABRE, in their promoter regions (Figure 5C; Supplementary Table 10). In pattern 6, 9 *PpZFPs* were specifically induced by early rehydration (PRE), and 13 dehydration-induced *PpZFPs* were phylogenetically clustered in group III and 6 in group II (Figure 5C; Supplementary Table 10). Of the dehydration-induced *AtZFPs*, only two and five clustered in patterns 2 and 6 (Figure 5D; Supplementary Table 10). Instead, pattern 4 included 17 and pattern 3 included 7 dehydration-induced *AtZFPs* (Figure 5D; Supplementary Table 10). These AtZFPs represented the specifically accumulated transcripts at the early stage of dehydration and full rehydration. Twenty of 36 dehydration-induced *AtZFPs* were phylogenetically clustered in group III and 10 in group IV (Figure 5D; Supplementary Table 10). In addition, the *PpZFPs* containing only the M-type C2H2-ZF domain(s) accounted for the dominant type among the differentially expressed *PpZFPs*, while the differentially expressed *AtZFPs* containing only the Q-type C2H2-ZF domain(s) ranged second after the *AtZFPs* containing only the M-type C2H2-ZF domain(s) (Figures 5C,D). These results might imply different regulatory mechanisms of C2H2-ZFPs during the responses to dehydration and rehydration between desiccation-tolerant *P. patens* and dehydration-sensitive *A. thaliana*.

### Subcellular localization of candidate key PpZFPs

**Figure 6:**
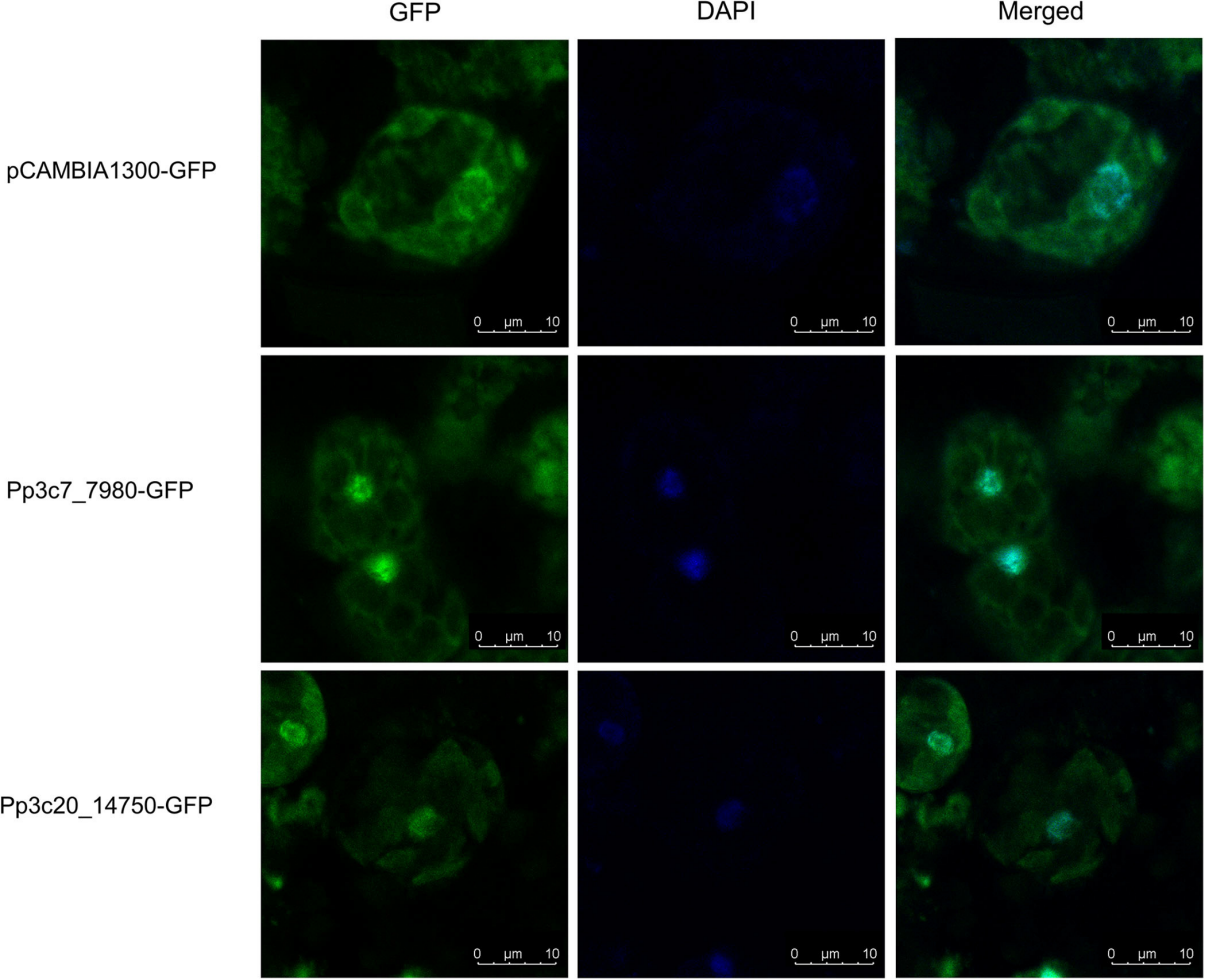
Subcellular localization of the key PpZFPs in the responses of dehydration and rehydration. Scale bar = 10 μM.

To further demonstrate the potential role of PpZFPs in the regulation of desiccation response, two candidate PpZFPs, *Pp3c7_7980* and *Pp3c20_14750*, which were specifically induced by severe dehydration, were selected to construct PpZFP-GFP fusion proteins for transient transformation to the protoplast of *P. patens*. The fluorescence of the two PpZFP-GFP fusion proteins and DAPI were detected to exclusively colocalize in the nuclei of the cells, whereas the fluorescence in the control cells only transformed by GFP construct was distributed in the whole cell parts except for the cell wall (Figure 6).

## Discussion

### Common and unique characteristics of C2H2-ZFPs in the major land plant lineages

C2H2-ZFPs, as plant-specific signaling agents for sense phytohormones, play important regulatory roles in the processes of various angiosperm plants from growth and development to environmental stresses (Han et al., 2020; Liu Y. T. et al., 2020). The development of whole-genome sequencing technology has facilitated the genome-wide identification of C2H2-ZFPs, but the identification mainly focused on some major and horticultural crops, as well as other economically important plants, except for the rice and *Arabidopsis* (Yuan et al., 2018a). It is rare to be reported in the non-seed plants, and the number of identified C2H2-ZPFs in *O. sativa* and *A. thaliana* varies in different reports (Englbrecht et al., 2004; Agarwal et al., 2007; Chen et al., 2020b). In this study, the C2H2-ZFPs lacking the typical C2H2-ZF domain are eliminated from the lists of C2H2-ZFPs in *A. thaliana* and *O. sativa*, resulting in the reidentification of 158 AtZFPs and 168 OsZFPs (Table 1). Based on the same reidentification criteria, the C2H2-ZFPs in four representatives of non-seed land plant lineages have also been determined for the first time, with 53 SmZFPs, 112 PpZFPs, 41 SfZFPs, and 17 MpZFPs (Table 1). Our analyses of the general characteristics of the representative species reveal that 548 of the identified C2H2-ZFPs displayed a hydrophilic nature, of which 534 could be located in the nucleus. The identified C2H2-ZFPs are classified into four great categories (named Q-, M-, Z-, and D-type) according to the variation of the conserved amino acid motif “QALGGH” and distances between metal ligands (Figure 2; Supplementary Table 1). These results reflect the conserved functions of C2H2-ZFPs among land plant lineages.

*Cis*-acting elements in the promoter region of genes are known to play important roles in the regulation of gene expression (Sharma et al., 2011). Our prediction reveals diverse cis-acting elements in the *C2H2-ZFP* promoters of the representatives of land plants that are functionally related to plant growth and development, phytohormone, biotic and abiotic stresses (Supplementary Table 7), whereas a large number of ABA- and/or drought-responsive elements (ABREs and DREs) dominate the identified *C2H2-ZFPs*. It should be mentioned that the majority of the predicted ABR elements are composite, including the core “ACGTG”, which is part of the larger CACGTG motif (Figure 3B). The motif “CACGTG”, initially described as a G-box, was first found in the promoter region of the light-regulated *rbcS* small subunit gene (Yu et al., 2014). It was later identified in the promoter regions of various functional and regulatory genes and often part of the promoters of light-induced genes, such as PIFs and PNZIP, involved in the responses to several stimuli (Yang et al., 2009; Leivar et al., 2012; Pedrotti et al., 2018; Zhang et al., 2019; Qi et al., 2020). In our prediction, ACGTG is not only part of G-box, but also part of CGCACGTGTC, CGTACGTGCA, and GCAACGTGTC (Supplementary Table 7). Therefore, we separate it out from the CACGTG in this study. These previous studies mean that the existence alone does not necessarily mean that the gene promoters we studied here are all regulated by dehydration. However, compared with non-seed plants, more ABRE and DRE elements are predicted in the promoters of *AtZFPs* and *OsZFPs*, suggesting the important roles of ABA and drought signals in C2H2-ZFP functions, and more precise regulation of *C2H2-ZFPs* by ABA and/or drought stress in seed plants. This result also indicates that the expression of *C2H2*-*ZFPs* could be partially induced by ABA-dependent behavior in responses to drought/dehydration stresses. However, whether these genes are involved in ABA-mediated drought response needs to be explored further. The results of this study provide not only the first comprehensive understanding of the genome-wide identification, but also the first systematic analyses of the structure, features, and potential functions of C2H2-ZFPs in major land plant lineages, especially non-seed plants.

### WGD events and evolution of C2H2-ZFPs in non-seed and seed land plants

Our results show that the representatives of major land plant lineages display an increase in C2H2-ZFP members from lycophyte (17 MpZFPs) to bryophyte (41 SfZFPs) to club moss (53 SmZFPs) to angiosperms (158 AtZFPs and 168 OsZFPs) with their evolutionary status. The only exception of the representatives among bryophytes is the model moss *P. patens*, whose genome encodes 112 PpZFPs, which is far more than that of other representatives of bryophytes, and *Lycopodium*. The significant expansion of AtZFPs, OsZFPs, and PpZFPs could be related to the whole-genome duplication events (WGD) during the evolution of their common ancestors. WGD events are ubiquity in organisms including plants, which provides an important source of raw materials for the expansion of gene family, and have long been regarded as important driving forces in speciation, adaptation, and diversification (Gu et al., 2003; Wood et al., 2009; Ohno, 2011; Soltis and Soltis, 2016; Wu et al., 2019). The previous study has revealed five rounds of polyploidy in *Arabidopsis*, including the ε event that occurred in the ancestor of angiosperms and ξ event in the ancestor for all seed plants (Blanc et al., 2003; Bowers et al., 2003; Jiao et al., 2011). The paleo-hexaploidy (γ) event occurred after the divergence of monocotyledons and dicotyledons and was followed by two WGDs (α and β) (Blanc et al., 2003; Bowers et al., 2003; Jiao et al., 2011). Echoing the WGD α*-*β*-*γ series, the ρ*-*σ*-*τ series was reported in grasses including rice (Jiao et al., 2014). *P. patens* also experienced a recent WGD event, which played an important role in the rapid expansion of the genome and gene families (Jenkins et al., 2018; Gao et al., 2022). Compared with *P. patens*, other non-seed plant representatives lacked recent WGD events (Jenkins et al., 2018; Gao et al., 2022), which likely accounts for the less member of *C2H2-ZFPs* in the other three representatives of non-seed plants.

In addition to WGD events, segmental duplication, tandem duplication, and transposition events are considered to represent three principal evolutionary patterns (Kong et al., 2007). Of these patterns, segmental and tandem duplications represent two of the main causes of gene family expansion in plants (Cannon et al., 2004). There are few syntenic regions between species with distant genetic relationship due to the great genome rearrangement, while the closely related species often contain abundant synteny (Jiao et al., 2014). No interspecies syntenic *C2H2-ZFPs* are detected between any two species of non-seed plants, and between any non-seed plant species and *A. thaliana* or *O. sativa*. This result is consistent with the relatively distant evolutionary and genetic relationships between representatives of land plant lineages. Intraspecies syntenic analysis revealed 14, 29, and 29 pairs of duplicated events in C2H2-ZFPs of *P. patens, A. thaliana*, and *O. sativa*, respectively, supporting the high conservation C2H2-ZFPs and the limited contribution of fragmental duplication to the family expansion. Further analysis of the selection pressure of all paired C2H2-ZFPs in the three species revealed a relatively low *Ka/Ks* ratio (< 0.5), except for three pairs OsZFPs with the *Ka/Ks* ratio >0.9 (Supplementary Table 4). This result indicates that most C2H2-ZFPs undergo strong purification selection in the evolutionary process. In addition, the lack of events in tandem duplication and segmental duplication of SmZEPs, SfZFPs, and MpZFPs, as well as the few tandem and segmental duplicated C2H2-ZFP pairs of PpZFPs, AtZFPs, and OsZFPs, suggests that WGD events but not the tandem and segmental duplications are the major driving force for the expansion of C2H2-ZFPs in land plant lineages.

The unique subgroups (Ib, IVa, and IVc) of AtZFPs and OsZFPs in the 549 C2H2-ZFPs-based phylogenetic tree indicate the differentiation and complexity of the functions of C2H2-ZFPs in seed plants. Previous reports indicated that analysis on gene structure can provide important clues for exploring the evolution of multi-gene families (Boudet et al., 2001; Babenko et al., 2004). Our integrated analyses of gene structures and conserved motifs and the phylogenetic relationships of the identified C2H2-ZFPs reveal a consistent trend on gene structure characteristics among the land plant representatives. For example, C2H2-ZFPs in the same group show similar exon and intron numbers and conserved domain type, and genes with large number of introns are located at the base of the phylogeny (such as *Pp3c25_12300* and *At5G51230*), indicating that genes with less introns evolved later. This supports the previous speculation that genes with a small number of introns may evolve in the way of intron loss, which may also be a way for plants to adapt to environmental stress (Zhu et al., 2016). In addition, syntenic gene pairs are all clustered in the same phylogenetic clade, which may reflect the conservation of the functions of *C2H2*-*ZFPs* in the same group. As a pioneer landing plant model and a desiccation-tolerant moss, *P. patens* genome encodes more PpZFPs belonging to group III, followed by those to group IV, while the number of AtZFPs belonging to group IV encoded by the dehydration-sensitive dicot *A. thaliana* is much higher than that of group III. The results indicate that *A. thaliana* and *P. patens* evolved their own unique adaptative strategies while gradually exploring the dramatic terrestrial environment.

### Expression profiles of PpZFPs and AtZFPs reveal the important roles and functional differences in responses to dehydration and rehydration

Water deficit is the first problem to be faced in the evolution of plants from aquatic to terrestrial. Majority of vascular plants adapt to water deficit by changing their architectures and cellular components, such as of root structure, vascular tissue, closable stoma, cuticle, and lignin accumulation. However, bryophytes, as the intermediate group between aquatic and land plants, can only adapt to dehydration at the cellular level due to the lack of these structures and tissues (Heckman et al., 2001). In addition, such dehydration tolerance is usually constitutive, which makes bryophytes have stronger dehydration resistance and the most primitive dehydration-resistant mechanism (Zhang et al., 2004). Previous studies have shown that ABA-related stomatal regulation and core ABA signaling components have been acquired in mosses and have been preserved during 400 million years of plant evolution (Chater et al., 2011). The release of the moss *P. patens* genome facilitates to identify and characterize important transcription factor (TF) genes, such as MYB and bHLH families, on a genome-wide scale (Rensing et al., 2008; Carretero-Paulet et al., 2010; Lang et al., 2018; Pu et al., 2020). Before this study, no genome-wide identification and characterization have been done with PpZFPs, and there has been no detailed study on whether and how its members participate in the regulation of dehydration and rehydration responses in the desiccation-tolerant *P. patens*. As a model of dicotyledons, the AtZFP family members and their functions in *A. thaliana* have been reported in many studies. However, it is still a lack of comprehensive investigation on the participation of AtZFPs in the responses to dehydration and rehydration.

Our expression profiles of *PpZFPs* and *AtZFPs* revealed different patterns in the responses to dehydration and rehydration, which are characterized by the differences in phylogenetic relationships, and types of conserved C2H2-ZF domains. Compared with 36 differentially expressed *AtZFPs* that are continuously induced during dehydration and rehydration, 11 out of the 26 dehydration-induced *PpZFPs* are specifically induced by dehydration, reached the peak abundance at SDH stage, and recovered their transcript abundance during rehydration (Pattern 2). Interestingly, the promoter regions of the 11 *PpZFPs* predicted the existence of ABRE *cis*-acting elements. On the one hand, the differences reflect the different response strategies of the desiccation-tolerant *P. patens* and the dehydration-sensitive *A. thaliana* to dehydration and rehydration. On the other hand, it also reflects that the phytohormone ABA could be the key regulator of the survival of the vegetative of *P. patens* under severe dehydration. Our results here combined with the previous published proteomic findings (Wang et al., 2010) enable us to propose that *P. patens* can quickly shut down the processes related to primary metabolism, growth, and development by downregulating related genes, thus protecting its cellular structures from damage and inducing the abiotic stress-responsive genes, but all these processes could be reversible once cellular water is recovered. While in *A. thaliana*, a positive-responsive strategy is adopted to fight water shortage by inducing large number of abiotic stress-related genes and physiological responses, followed by gradually shutting down basic metabolism. However, the plants cannot completely recover due to the severe damage of cellular structures. A previous study revealed that *AZF1* (*AT5G67450*) and *AZF2* (*AT3G19580*), two transcription inhibitors in *A. thaliana*, negatively regulated plant growth by repressing the expression of osmotic stress and ABA inhibitory genes (Kodaira et al., 2011). Another AtZFP encoding gene in *A. thaliana, ZAT18* significantly improved the plant drought tolerance of transgenic plants by positively regulating drought response (Yin et al., 2017). The transgenic plants of *ZAT7* (*AT3G46090*) displayed salt-tolerant phenotypes (Ciftci-Yilmaz et al., 2007). In the present study, the expression pattern of these three genes induced by dehydration and rehydration also supports the previous reports on their functions.

In the past decades, although a large number of C2H2-ZFPs have been identified in the genomes of many model plants and crops, functional analysis has mainly focused on the studies of the Q-type C2H2-ZFPs (Kam et al., 2008; Gourcilleau et al., 2011; Kim et al., 2013; Wang et al., 2013, 2016; Muthamilarasan et al., 2014; Cheuk and Houde, 2016; Lawrence and Novak, 2018; Liu Y. T. et al., 2020). These previous studies indicated that the Q-type C2H2-ZFPs are involved in a wide range of biological processes, including development and organogenesis, as well as responses to stresses and defense in model angiosperms. In our present study, 7 Q-type *PpZFPs* (3 upregulated and 4 downregulated) and 15 Q-type *AtZFPs* (8 upregulated and 7 downregulated) responded to dehydration and rehydration, respectively (Figures 5C,D). These results suggest the functional conservation of Q-type C2H2-ZFPs among land plants. Interestingly, the M-type *C2H2-ZFPs* with the largest number followed by the Z-type constitute the main types of *P. patens* and *A. thaliana* in responses to dehydration and rehydration (Figures 5C,D). However, there are rare reports on the functions of the two type *C2H2-ZFPs* in abiotic stress responses, especially in responses to dehydration and desiccation. Therefore, the in-depth investigation of dehydration-induced M-type and Z-type PpZFPs and AtZFPs will cast new light on their functions and evolution among land plants.

Taken together, our work in this study highlights the conservation in structure, function, and evolution of C2H2-ZFPs in major land plant lineages and the importance of C2H2-ZFPs in dehydration tolerance of *P. patens* and *A. thaliana*. This study is also the first time to identify the C2H2-ZFPs genes and examine their expression patterns in *P. paten*s. Further characterization of the PpZFPs specifically induced by severe dehydration identified here will shed light on an ABA-responsive mechanism that could be unique to the nonvascular plant *P. patens*. The findings here lay the foundations for further functional research of C2H2-ZFP genes in dehydration responses from an evolutionary perspective in land plants and provide us with potential targets for drought tolerance breeding in crops and beyond.

## Data availability statement

The data presented in the study are deposited in the NCBI repository, accession number BioProject: PRJNA800025.

## Author contributions

LX conceived the study. XL and LX designed the experiments and wrote the manuscript. XL, XC, and LX performed the bioinformatic analyses. XL, XC, JL, QN, and YM prepared the plant materials and conducted the experiments. All authors contributed to the article and approved the submitted version.

## Funding

This study was supported by grants from the National Natural Science Foundation of China (Grant No. 31970288) and the Research and Development Fund of Zhejiang A&F University (Grant No. 2018FR002).

## Conflict of interest

The authors declare that the research was conducted in the absence of any commercial or financial relationships that could be construed as a potential conflict of interest.

## Publisher's note

All claims expressed in this article are solely those of the authors and do not necessarily represent those of their affiliated organizations, or those of the publisher, the editors and the reviewers. Any product that may be evaluated in this article, or claim that may be made by its manufacturer, is not guaranteed or endorsed by the publisher.
