# Supplementary material for: Genome-wide characterization of C2H2 zinc-finger gene family provides insight into the mechanisms and evolution of the dehydration–rehydration responses in Physcomitrium and Arabidopsis
Source: Front Plant Sci. 2022 Oct 3;13:953459. doi: 10.3389/fpls.2022.953459 (PMC9574186; doi:10.3389/fpls.2022.953459)

Supplementary Table 1. RNA-Seq information of *P. patens* and *A. thaliana* during dehydration and rehydration.

Supplementary Table 2. Primers for the constructs of transient subcellular localization of two key *PpZFPs*.

Supplementary Table 3. Identified C2H2-ZFPs in the representatives of land plant lineages.

Supplementary Table 4. Syntenic paralogs and the *Ka/Ks* ratios of *C2H2-ZFPs* in *P. patens*, *A. thaliana* and *O. sativa.*

Supplementary Table 5. General characteristics of C2H2-ZFPs of the representatives of land plant lineages.

Supplementary Table 6. The C2H2-ZF and other known domains besides in each analyzed species.

Supplementary Table 7. Predicted elements in the promoter regions upstream the start codom of C2H2-ZFPs in the representatives of land plant lineages.

Supplementary Table 8. Aligned C2H2-ZFs of the C2H2-ZFPs used for phylogenetic analysis.

Supplementary Table 9. Details for genes in each group of phylogeny containing PpZFPs and AtZFPs.

Supplementary Table 10. Profiles and details of the differentially expressed *PpZEPs* and *AtZEPs*.

Supplementary Figure 1. Vector construction for 35S::Pp3c2_14750 and 35S::Pp3c7_7980 fusion proteins. The backbone is pCAMBIA1300 binary vector.

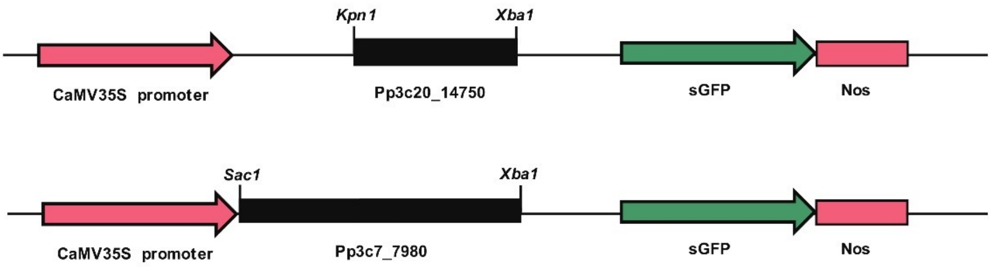

Supplementary Figure 2. Genomic localization and synteny of AtZFPs in *A. thaliana*. The scale is shown in Mb at the left of the figure and the chromosome numbers are located at the top of the chart. The tandem repeated genes are marked by red color on the chromosomes.

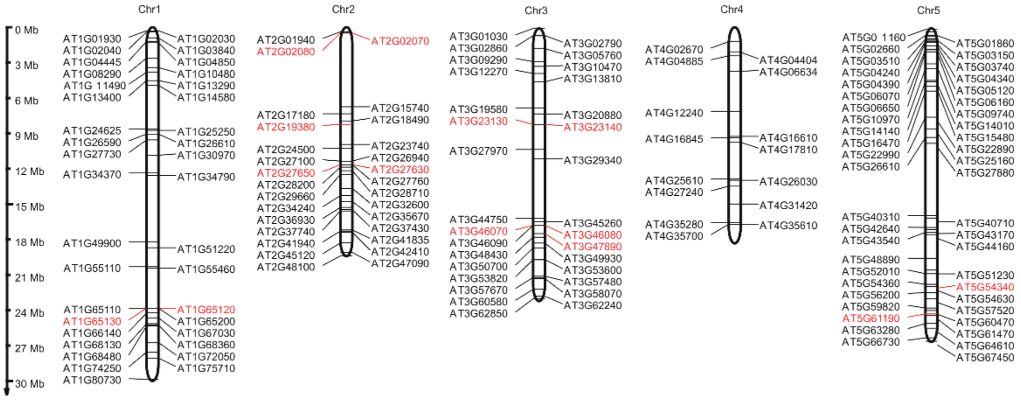

Supplementary Figure 3. Genomic localization and synteny of OsZFPs in *O. sativa*. The scale is shown in Mb at the left of the figure and the chromosome numbers are located at the top of the chart. The tandem repeated genes are marked by red color on the chromosomes.

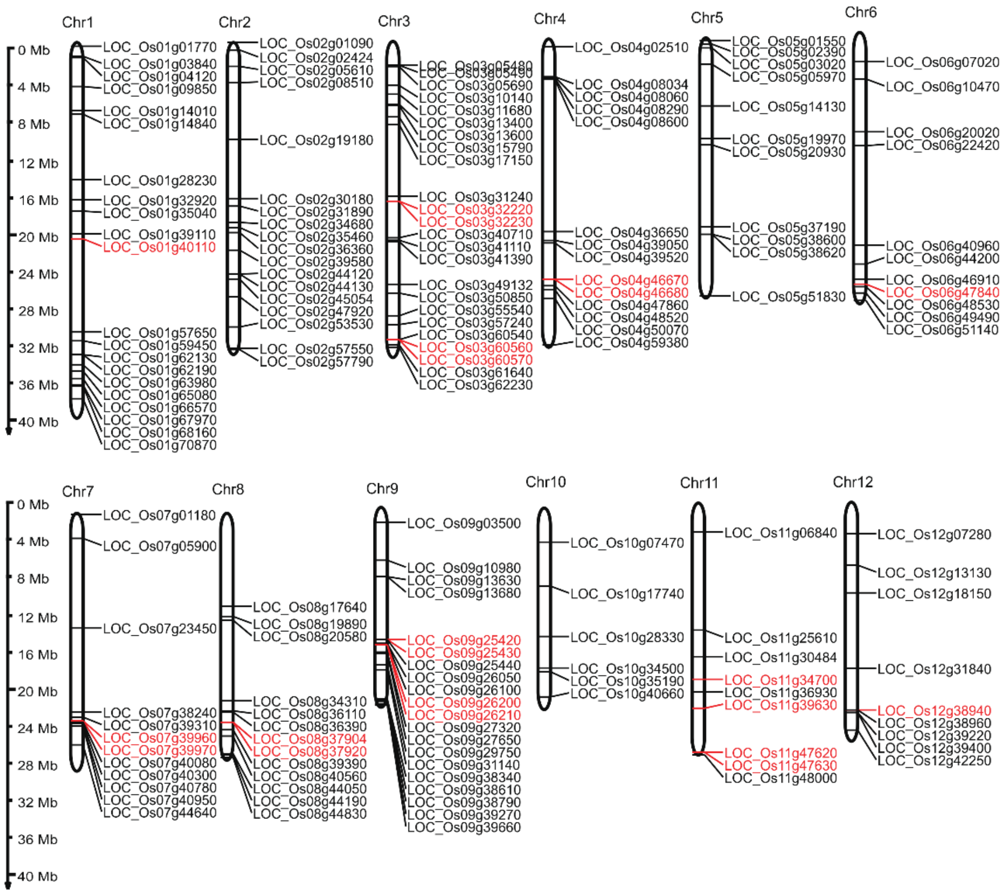

Supplementary Figure 4. Genomic localization and synteny of PpZFPs in *P. patens*. The scale is shown in Mb at the left of the figure and the chromosome numbers are located at the top of the chart. The tandem repeated genes are marked by red color on the chromosomes.

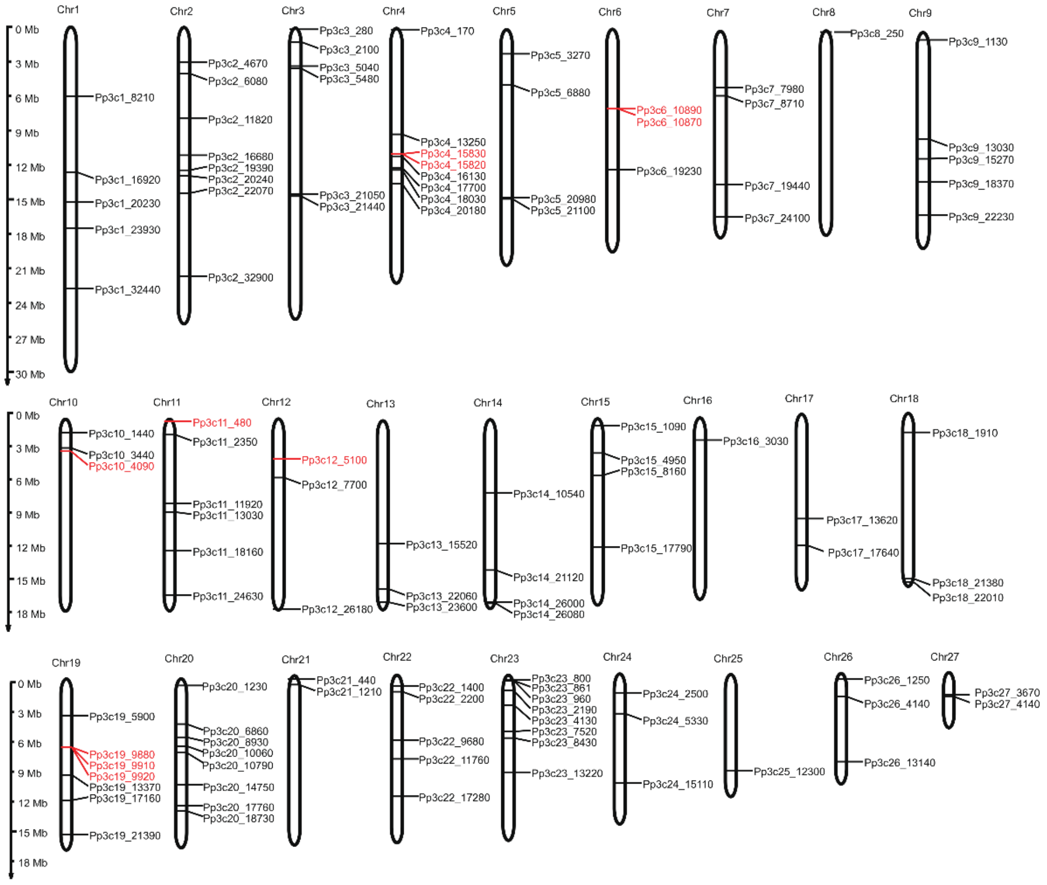

Supplementary Figure 5. Genomic localization and synteny of SfZFPs in *S. fallax*. The scale is shown in Mb at the left of the figure and the chromosome numbers are located at the top of the chart. The tandem repeated genes are marked by red color on the chromosomes.

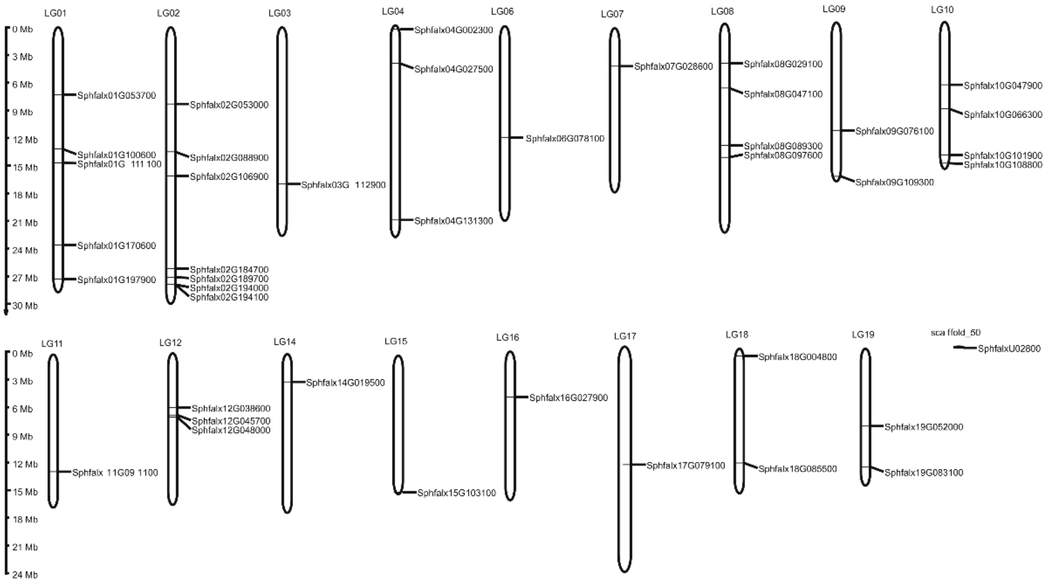

Supplementary Figure 6. Genomic localization and synteny of SmZFPs in *S. moellendorffii*. The scale is shown in Mb at the left of the figure and the chromosome numbers are located at the top of the chart. The tandem repeated genes are marked by red color on the chromosomes.

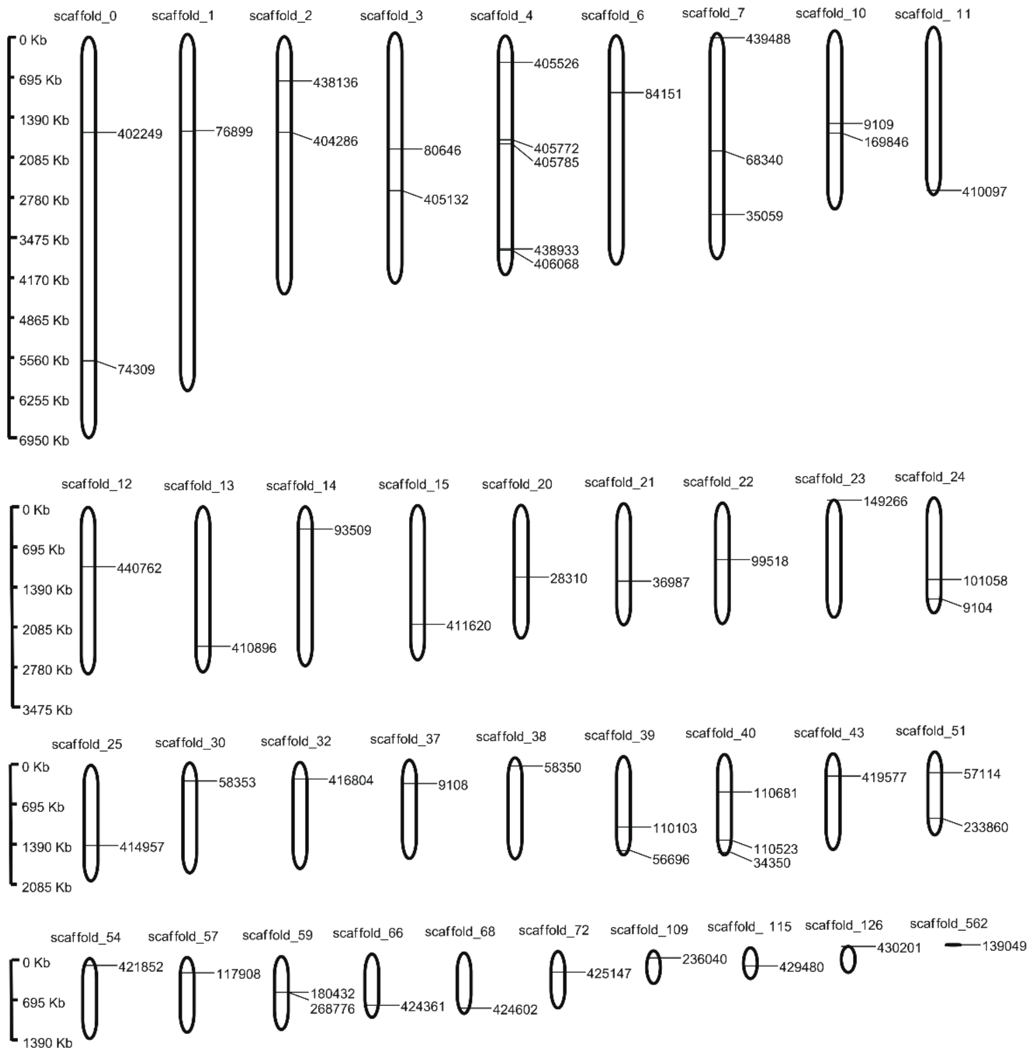

Supplementary Figure 7. Genomic localization and synteny of MpZFPs in *M. polymorpha*. The scale is shown in Mb at the left of the figure and the chromosome numbers are located at the top of the chart. The tandem repeated genes are marked by red color on the chromosomes.

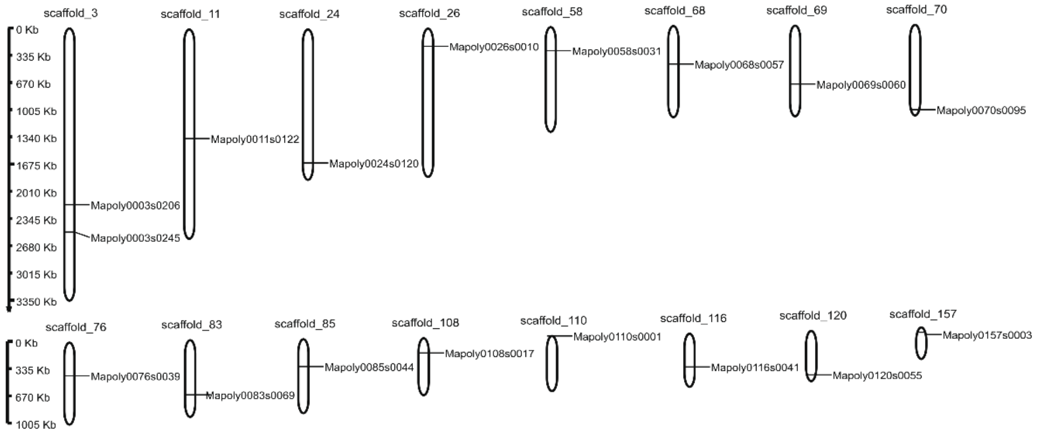

Supplementary Figure 8. Gene structure of AtZFPs in *A. thaliana*. The yellow and blue rectangles represent the CDSs and UTRs respectively, and the black lines represent introns.

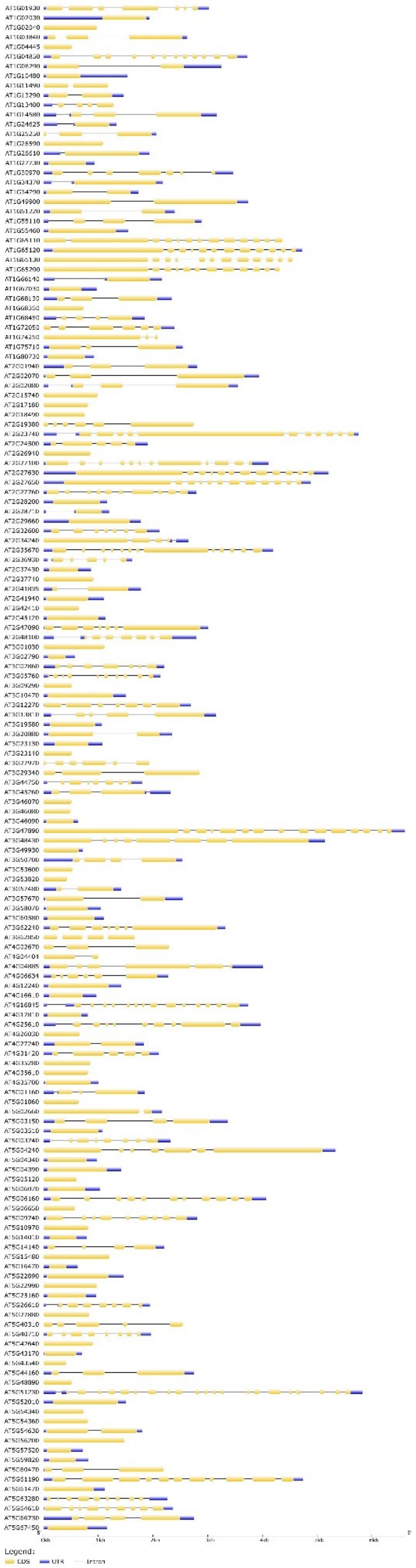

Supplementary Figure 9. Gene structure of OsZFPs in *O. sativa*. The yellow and blue rectangles represent the CDSs and UTRs respectively, and the black lines represent introns.

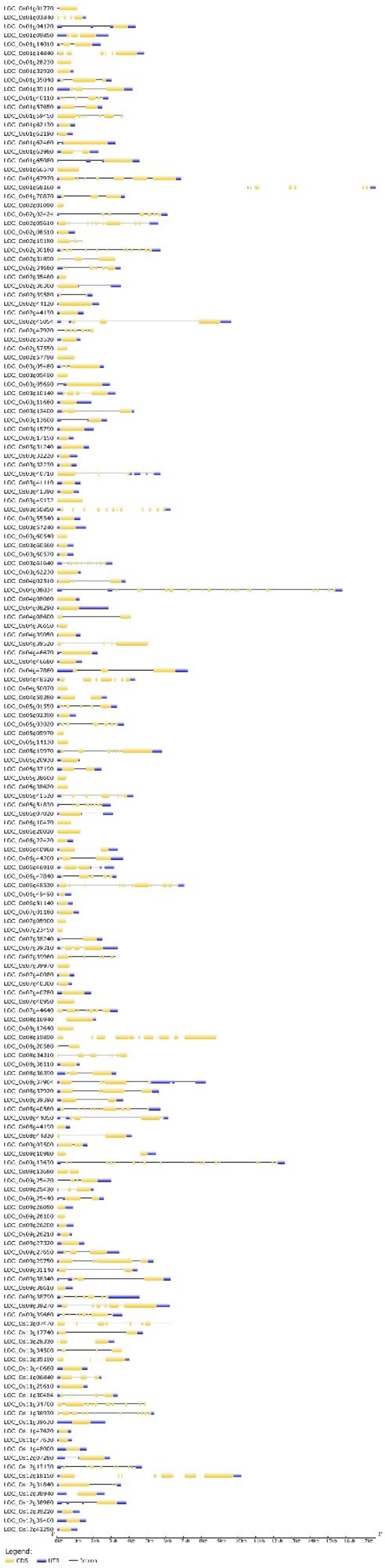

Supplementary Figure 10. Gene structure of PpZFPs in *P. patens*. The yellow and blue rectangles represent the CDSs and UTRs respectively, and the black lines represent introns.

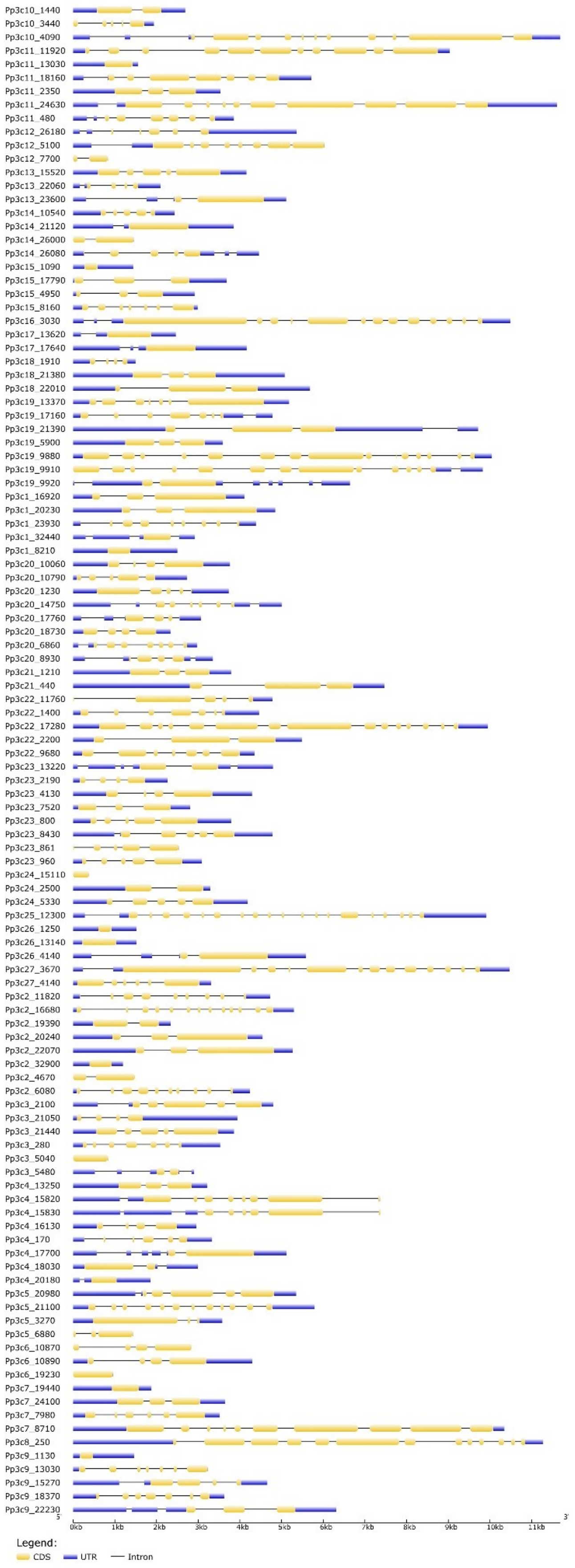

Supplementary Figure 11.Ggene structure of SfZFPs in *S. fallax*. The yellow and blue rectangles represent the CDSs and UTRs respectively, and the black lines represent introns.

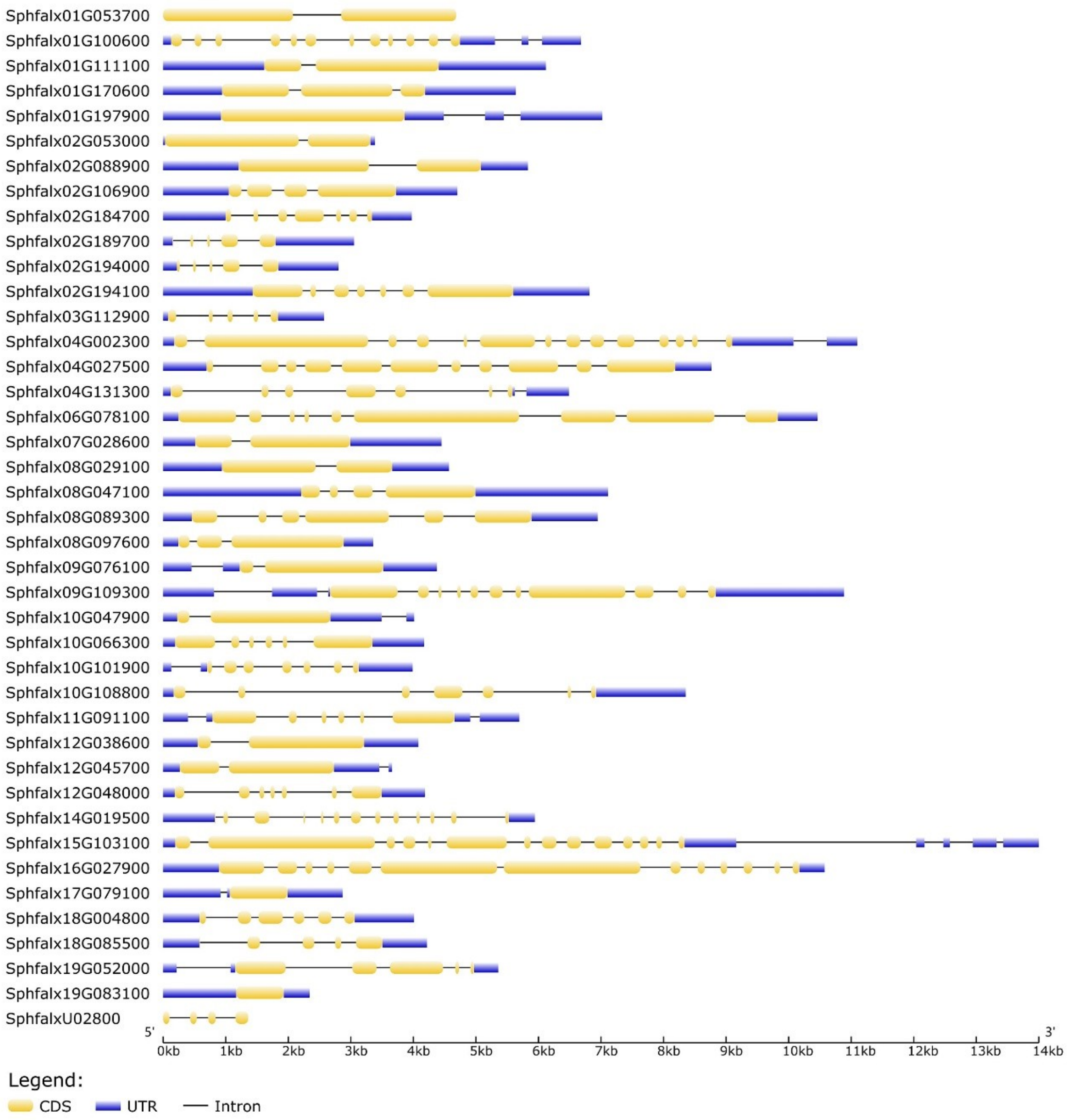

Supplementary Figure 12. Gene structure of SmZFPs in *S. moellendorffii*. The yellow and blue rectangles represent the CDSs and UTRs respectively, and the black lines represent introns.

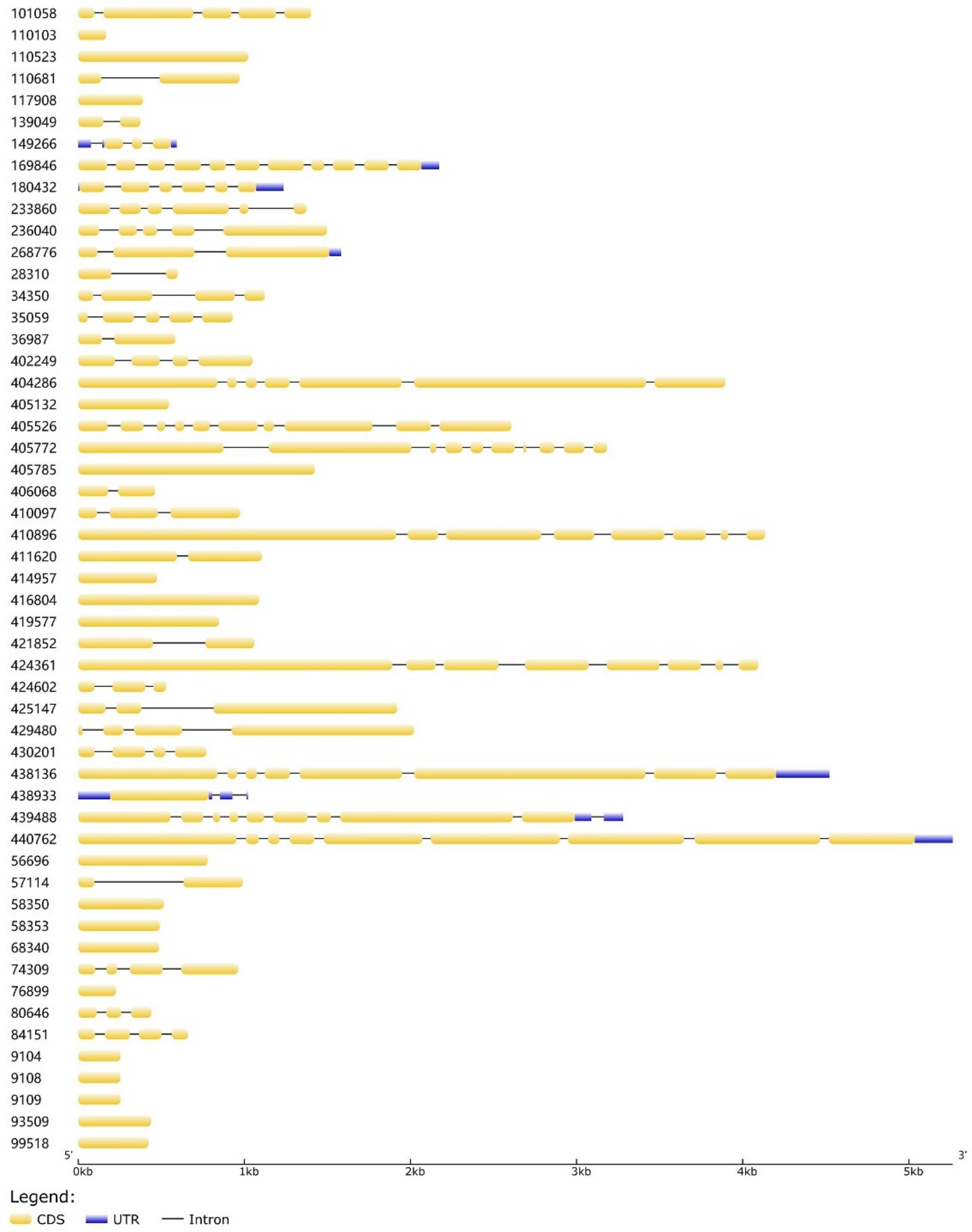

Supplementary Figure 13. Gene structure of MpZFPs in *M. polymorpha*. The yellow and blue rectangles represent the CDSs and UTRs respectively, and the black lines represent introns.

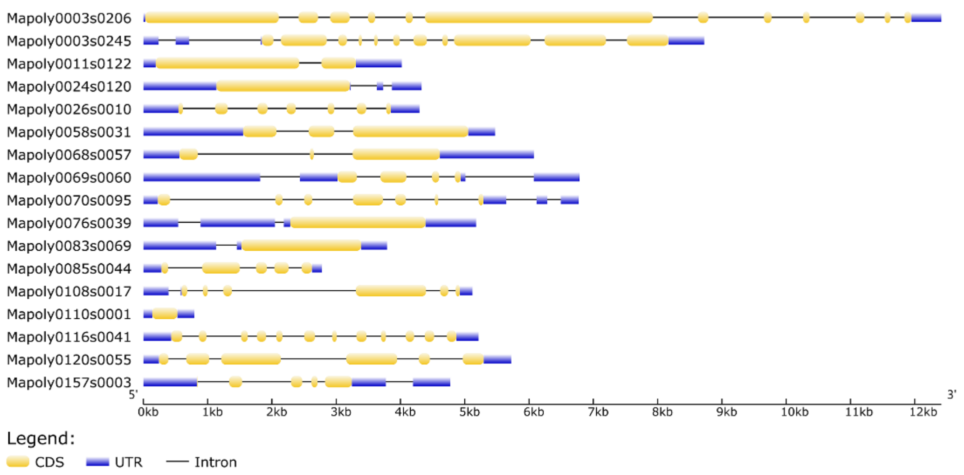

Supplementary Figure 14. The detailed phylogeny of C2H2-ZPFs in land plant representatives. The tree was constructed by iqtree 1.0 software using Maximum Likelihood (ML) method with 1000 bootstraps. The C2H2-ZFPs containing only C2H2 domain are marked with small red circles.

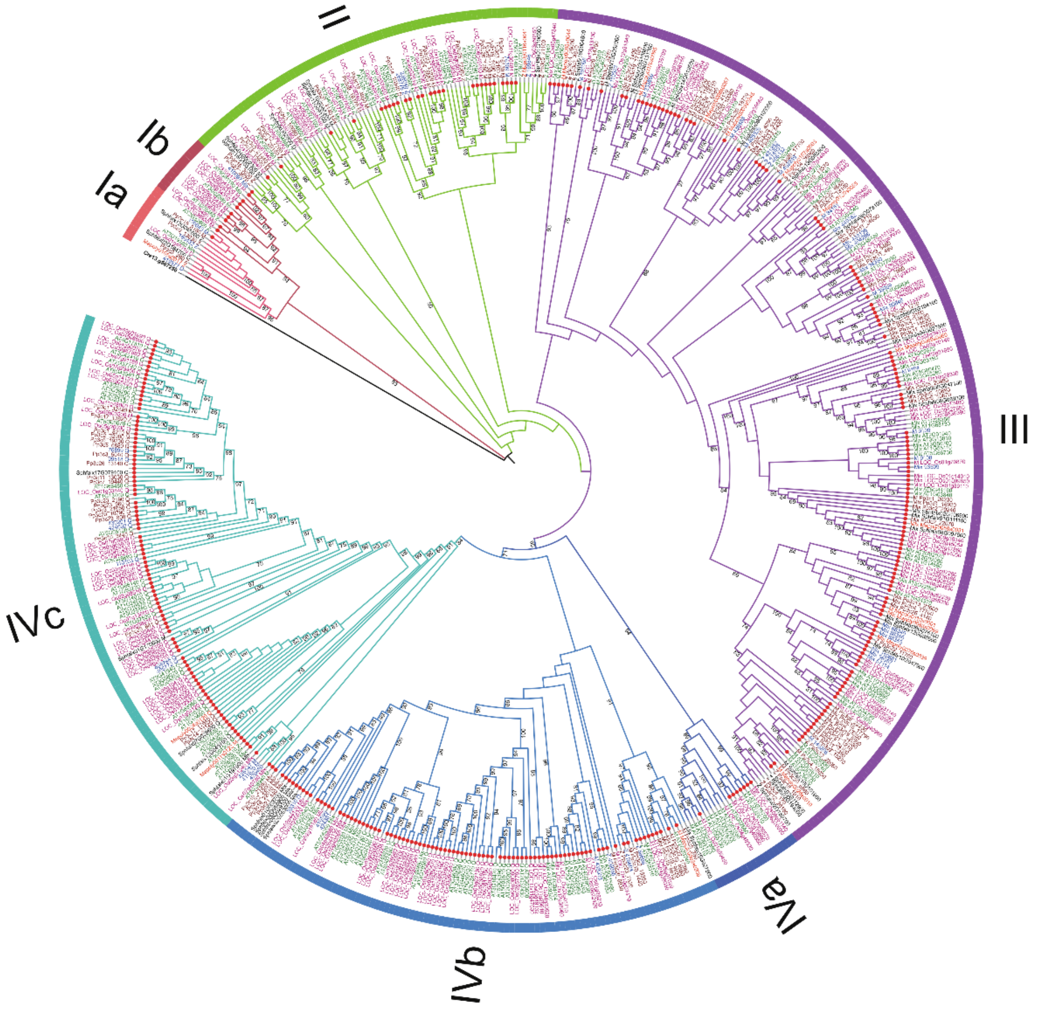

Supplementary Figure 15. The detailed phylogeny of AtZPFs in *A. thaliana*. The tree was constructed by iqtree 1.0 software using Maximum Likelihood (ML) method with 1000 bootstraps. The C2H2-ZFPs containing only C2H2 domain are marked with small red circles.

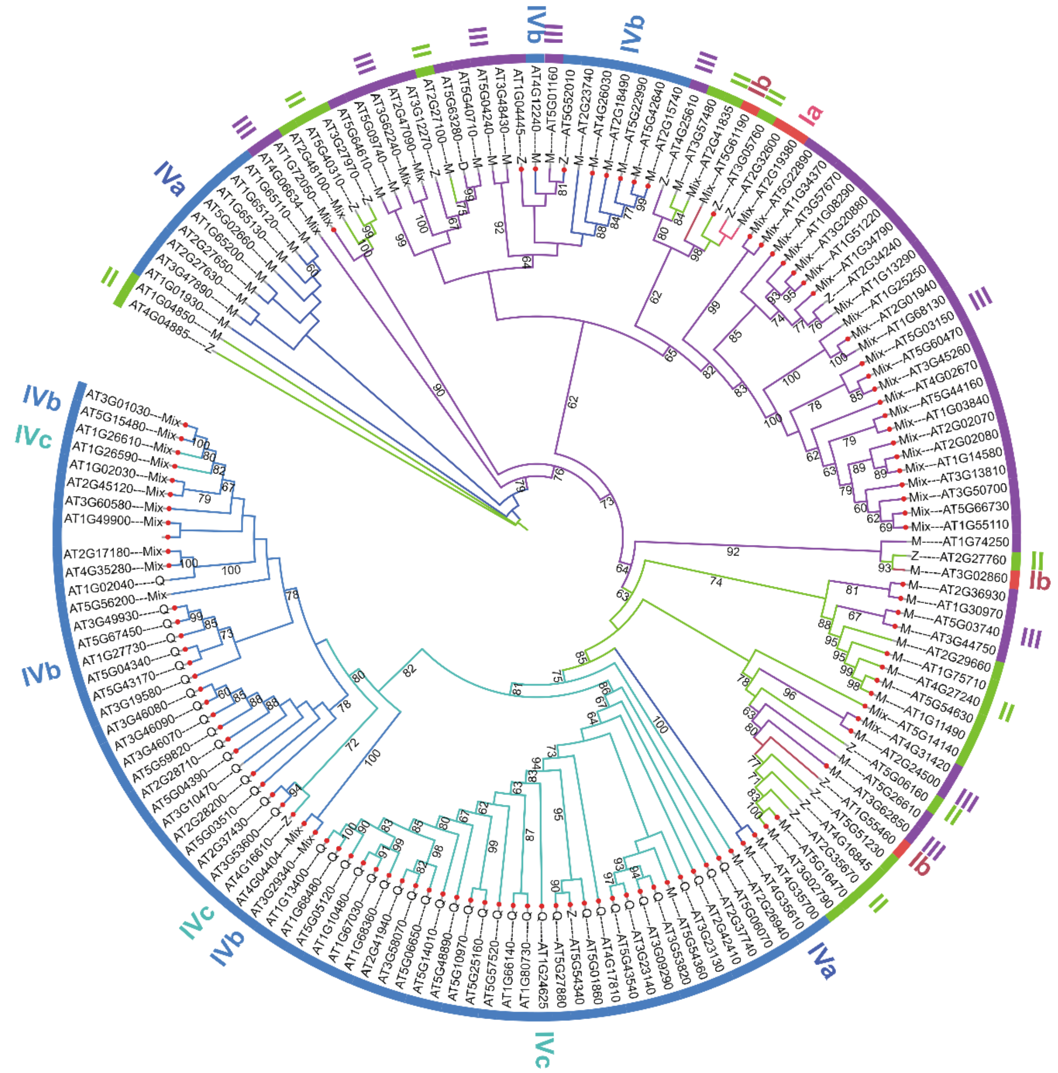

Supplementary Figure 16. The detailed phylogeny of PpZFPs in *P. patens*. The tree was constructed by iqtree 1.0 software using Maximum Likelihood (ML) method with 1000 bootstraps. The C2H2-ZFPs containing only C2H2 domain are marked with small red circles.

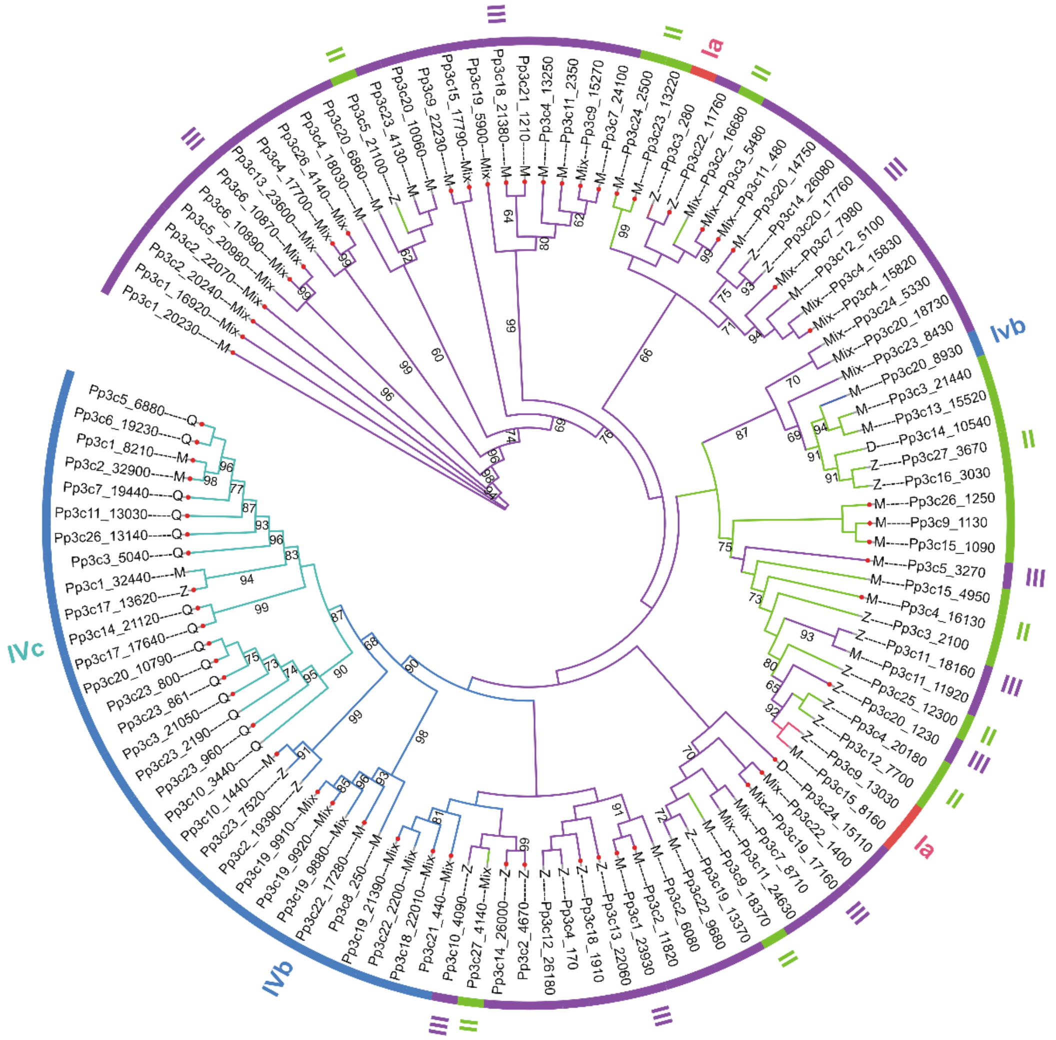

Supplement: Supplementary file 1 [file Data_Sheet_1.PDF]
